# Response of DNA methylation and gene expression to light treatments in Norway spruce [*Picea abies* (L.) Karst.]

**DOI:** 10.1186/s12864-026-12877-7

**Published:** 2026-05-06

**Authors:** Fangqun OuYang, Jinping Zhang, Ran He, Junhui Wang, Mulualem Tigabu, Jianxun Luo

**Affiliations:** 1 Beijing Floriculture Engineering Technology Research Centre, Key Laboratory of National Forestry and Grassland Administration on Plant Ex situ Conservation, Beijing Botanical Garden, Beijing, 100093 China; 2 School of Mathematics and Physics, North China Electric Power University, Beijing, 102206 China; 3 State Key Laboratory of Tree Genetics and Breeding, Key Laboratory of Tree Breeding and Cultivation of State Forestry Administration, Research Institute of Forestry, China Academy of Forestry, Beijing, China; 4 College of Forestry, Fujian Agriculture and Forestry University, Fuzhou, 350002 China; 5 Sichuan Academy of Forestry, Chengdu, Sichuan China

**Keywords:** Differentially methylated regions, DNA methylome, Far-red light, Light intensity, *Picea abies*, Transcriptome

## Abstract

DNA methylation is important for many developmental processes. Its role in plant’s adaptation to light conditions, however, remains unclear. In this study, we examined the DNA methylomes of Norway spruce (*Picea abies* (L.) Karst.) under different light conditions, a combination of both light intensities (10, and 80 µmolm^−2^s^− 1^) and light qualities (with or without far-red light). The result showed that both light intensity and quality affected seedling growth, with the former playing a major role. Both light intensity and quality drove genome-wide methylation changes, which varied among cytosine contexts (CG/CHG vs. CHH). The changes in light quality and intensity yielded a similar number of differentially methylated regions (DMRs), but light intensity was a major contributor in gene expression changes. At the genome-wide level, DNA methylation changes showed no consistent correlation with gene expression alterations. However, a subset of genes—particularly those involved in photosynthesis and circadian rhythms—exhibited coordinated methylation and expression changes, suggesting context-specific regulatory links. The findings indicate that DNA methylation may directly modulate photosynthesis and photoperiod genes and trigger an epigenetic memory, which in turn affects growth of Norway spruce seedlings in response to light conditions. In sum, our study provides a global picture of DNA methylation and gene expression under different light conditions in conifers and thus paves a way for future studies of light-directed gene regulation in plants.

**Supplementary Information:**

The online version contains supplementary material available at 10.1186/s12864-026-12877-7.

## Introduction

Light is the energy source of photosynthesis for plants, but also a signal to activate photoreceptors (phytochrome, cryptochrome, phototropin and UVB-receptor), controlling gene expression, protein synthesis, and cell metabolism through signal transmission [1–4]. Thus, light plays a central role in major plant developmental processes, such as photomorphogenesis, photoperiodic induction of flowering, phototropism, and shade avoidance [5–10]. The natural environments often provide constantly fluctuating light conditions (varying intensity and quality), leading to excitations of two photosystems; photosystem I [PSI] and photosystem II [PSII] to varying extents [11, 12]. In order to maximize photosynthesis efficiency, plants are capable of making transitions between PSI and PSII to absorb different excitation energies. In the reversible regulation process, a balanced energy absorption is achieved by chloroplast protein kinase *Stt7* and phosphatase *TAP38* that regulate the phosphorylation of antenna complex LHCII [13]. Furthermore, the variable size of the PSII-LHCII supercomplex [14, 15] and the changing binding position of the peripheral light complex in the PSII-LHCII supercomplex regulate the light capture in the low light intensity and quench excess energy in high light intensity to achieve phenotypic reprograming [16].

DNA methylation is now well established as an important epigenetic marker due to its role in gene stability, transposable element (TE) silencing, chromosomal interactions, and transcriptional activity, development, and environmental responses [17]. In plants, DNA methylation occurs not only in the symmetric CG and CHG sequence contexts but also in the asymmetric CHH sequence context (H represents A, T, or C) [18–20]. Recent discoveries indicate that dynamic DNA methylation upon light changes is very important for stem elongation. For instance, a low red/far-red light ratio led to a lower level of methylation in *Stellaria longipes*, which was crucial for controlling the stem elongation response [21]. In maize, the promoter of PEPC is heavily methylated in roots and leaves, and the de-methylation of the region is dependent on light and is coincident with elevated PEPC expression [22]. In cotton, DNA methylation in *CONSTANS*-*LIKE* (*COL*) genes was found to be responsible for the loss of photoperiod sensitivity during cotton domestication, leading to delayed flowering [23]. In rice, high methylation of a long noncoding RNA resulted in male sterility under long day conditions [24]. Genome hypermethylation was detected in *Posidonia oceanica* when subjected to low-light conditions, including the methylation of light perception and harvesting genes such as *PHYB* and *LHCB5* [25]. In tree species, DNA methylation in the regulation of gene expression responses to abiotic environmental stresses, such as droughts [26, 27], low temperature [28, 29], salt stress and heavy metals [30, 31], was studied. However, DNA methylation in the regulation of light responses in conifers is largely unexplored.

Norway spruce (*Picea abies* (L.) Karst.) is one of the most widespread, ecologically and economically important conifers in Europe, and is the first gymnosperm that has been completely sequenced with a ~ 20-Gb genome [32]. Photoperiod was thought to be the primary factor controlling shoot elongation in Norway spruce, and thus extended-day treatments with different light qualities [33–35] and light intensities [36] have been explored. However, the effect of far-red light on seedling growth in Norway spruce was inconclusive [37, 38]. DNA methylation in response to different light conditions in Norway spruce is largely unexplored. Thus, this information is imperative for understanding how far-red light and light intensity affect seedling growth in Norway spruce using DNA methylation and transcriptome sequencing.

So far, the studies of epigenetic regulation by light have been focused on angiosperm, while studies in gymnosperm are rare, particularly at the methylome level. It has been known that gymnosperms and angiosperms have different mechanisms for light response [39, 40], and current knowledge about the regulation of photomorphogenesis is largely based on angiosperm model plants. Thus, in this study, we aim to reveal how DNA methylation changes under different light conditions in a gymnosperm plant, i.e., Norway spruce. Previous methylation studies focused on the effect of the epitype-inducing temperature conditions on DNA methylation changes in Norway spruce [41] and the effects of DNA methylation reconfiguration in bisexual cones on the expression of key genes in cone development in *Picea crassifolia* [42]. However, how DNA methylation contributes to the light response in conifers remains unclear. We hypothesized that high light intensity and a mixture of red and fared light induce more DNA methylation than low light intensity and monochromatic red light, which in turn activates expression of some genes and trigger epigenetic memory affecting growth of Norway spruce seedlings. We expect that DNA methylation will be accompanied by expression of genes related to photosynthesis and photoperiod, which are governed by light intensity and quality.

## Materials and methods

### Plant materials and growth conditions

Seedlings were grown from seeds of *P. abies* bought from the Center of Tree Seed of Canada (https://www.nrcan.gc.ca/science-data/research-centres-labs/forestry-research-centres/atlantic-forestry-centre/national-tree-seed-centre/13449), sourced from Natural Resources and Energy forests, with a provenance of 840 01 Norddeutsches Tiefland, collected in Mecklenburg-Vorpommern, Germany. When 80% seeds were dehiscent, the seeds were sown into plastic containers (5 cm in diameter and 5 cm in height), one seed per container. The growing medium consisted of a mixture of rural soil, peat, and perlite (containing 0.5 kg/m^3^ mix of zinc and phosphorus and 1.5 kg/m^3^ ferrous sulfate) in the ratio 3:2:1. After the hypocotyl grew to an average length of 1.99 cm, but before the cotyledons were completely open, one-month old seedlings were transferred to a Percival growth cabinet (LED-30 Elite series), which allowed controlling light conditions. In total, we established seven light treatments: red-light with low intensity (R10, R, 10 µmolm^−2^s^− 1^), red-light with middle intensity (R40, R, 40 µmolm^−2^s^− 1^), red-light with high intensity (R80, R, 80 µmolm^−2^s^− 1^), low intensity red light with far-red light (RFR10, R, 8.75 µmolm^−2^s^− 1^ plus FR, 1.25 µmolm^−2^s^− 1^), mid-intensity red light with far-red light (RFR40, R, 35 µmolm^−2^s^− 1^ plus FR, 5 µmolm^−2^s^− 1^), high intensity red light with far-red light (RFR80, R, 70 µmolm^−2^s^− 1^ plus FR, 10 µmolm^−2^s^− 1^), and mid-density white fluorescent lamp light (W40, W, 40 µmolm^−2^s^− 1^) as a control. These light conditions are similar to conditions used by other studies in angiosperms [43, 44], thus making comparisons feasible. For each light treatment, 120 seedlings (three replicates of 40 seedlings) were planted and watered every three days. The humidity was 75%. The light was arranged in the cycle of 16-hour light/8-hour dark (25˚C/15˚C) for 120 days.

### Measurement of seedling morphology

After light treatment for 120 days, the height (0.1 cm precision) and fresh biomass (0.01 g precision) of 20 randomly selected seedlings from each treatment were measured. After recording the measurements, the needles of the seedlings were frozen in liquid nitrogen and stored at -80℃ for later use.

### Total RNA isolation and RNA-seq library construction

We chose the four treatments, R10, RFR10, R80, RFR80 for RNA-seq and MethylC-Seq library generation to examine the effects of light intensity and quality on DNA methylation. The total RNA from the needles was extracted using OmniPlant RNA Kit, DNase I (Cwbiotech, Beijing, China), according to the manufacturer’s instructions. For each light treatment, three independent replicates (five seedlings randomly selected and pooled per replicate) were prepared and each replicate consisted of a mixture of five seedlings. The integrity and quality of each total RNA sample was determined by using NanoDrop 1000 Spectrophotometer and formaldehyde-agarose gel electrophoresis. Moreover, we only used RNA samples with the Abs260 nm/Abs280 nm ratio greater than 1.8 for downstream experiments.

The rRNA was removed from the total RNA by Ribo-ZeroTM Magnetic Kit (Epicentre, Madison, WI), and sequencing libraries were prepared by using the TruSeq RNA sample prep kit (Illumina) and sequenced on Illumina HiSeqTM 2500 (Illumina, San Diego, CA) by Gene Denovo Biotechnology Co. (Guangzhou, China).

### Mapping of RNA-seq reads, transcript assembly, and abundance estimation

The sequenced reads were first trimmed to removing adapters and low quality bases (Q scores < 28) by Trimmomatic (v 0.36) [45] and rRNA -derived reads were aligned against ribosome RNA (rRNA) database by using Bowtie2 (v 2.2.3) [46] and removed. The resulted reads were then mapped to reference genome of *P. abies* (http://congenie.org/) by TopHat2 (v 2.0.13) [47] with the following setting: 1) maximum read mismatch is 2;2) the distance between paired reads is 50 bp༛3) the deviation of the distance is 80 bp. The resulted alignments were used to create a reference annotation based transcripts (RABT) assembly using Cufflinks (v 2.2.1) [48] in the discovery mode, allowing detection of novel genes and splicing isoforms. The RABT assemblies were then merged across replicates and treatments by using Cuffmerge to get a comprehensive set of transcripts for downstream analyses. The expression level of each transcript was estimated using RSEM (v 0.6) [49] and expressed as Fragments Per Kilobase of transcript per Million mapped reads (FPKM). Finally, differentially expressed genes (DEGs) among treatments were identified by using edgeR [50] (https://bioconductor.org/packages/release/bioc/html/edgeR.html) with a fold change ≥ 2 and a false discovery rate (FDR) < 0.05.

DEGs were then subjected to enrichment analysis in Gene Ontology (GO, http://www.geneontology.org/) by using agriGO software (v 2.0) [51] and in Kyoto Encyclopedia of Genes and Genomes (KEGG) pathways by using KOBAS (v 3.0) [52]. GO terms and pathways that contained > 5 DEGs with q-values < 0.05 were considered significant. Principal component analysis (PCA) was performed by using the R package gmodels [53], (https://CRAN.R-project.org/package=gmodels*).*

### Genomic DNA extraction and MethylC-seq library generation

After genomic DNA (gDNA) was extracted from the needles using the Plant Genomic DNA kit2 (TIANGEN), DNA concentration and integrity were evaluated by NanoDrop spectrophotometer and Agarose Gel Electrophoresis, respectively. To prepare MethylC-seq libraries, we used 600 ng of purified gDNA, spiked in with 0.5% (wt/wt) unmethylated Lambda DNA (Promega, Madison, WI). There were three independent replicates per light treatment (five seedlings randomly selected and pooled per replicate). The gDNA were fragmented into 100–300 bp by Sonication (Covaris) and purified with MiniElute PCR Purification Kit (QIAGEN). The blunt fragmented DNAs were end-repaired by adding a single “A” nucleotide to the 3’ end, followed by ligated to methylated sequencing adapters. Fragments with adapters were bisulfite converted using Methylation-Gold kit (ZYMO) and then PCR amplified and sequenced on Illumina HiSeqTM 2500 by Gene Denovo Biotechnology Co. (Guangzhou, China).

### MethylC-seq data analysis

Raw reads were filtered by removing those having more than 10% of unknown nucleotides (N) or containing more than 40% of low quality (Q-value ≤ 20) bases. The filtered reads were mapped to *P. abies* genome using BSMAP software (v 2.90) [54] with the default setting. To simplify computation, we concatenated small Norway spruce scaffolds into 115 superscaffolds (average size 70 Mb) by inserting 200“N”bases between the original scaffolds. Only scaffolds longer than 3 kb or containing gene models were used for concatenation. This procedure was performed purely to improve computational efficiency and did not reflect any biological order or chromosomal arrangement. Scaffolds not meeting these criteria were retained as individual sequences and included in all subsequent analyses, with no loss of genomic information. Then methylated cytosines were called with a custom Perl script based on bismark (v0.19.0) and tested with the correction algorithm [55]. The methylation level at each cytosine was calculated as the percentage of reads supporting methylation among the total of sequenced reads. To assess different methylation patterns in different genomic regions such as gene body (or transposable elements) and at its flanking 2 kb regions, the methylation profiles were computed by averaging the methylation levels in each 100 bp interval.

To identify differentially methylated cytosines (DMCs), we required a read depth of at least 4. And for each cytosine context (CG, CHG and CHH), we have the following criteria [56]: 1) For CG and CHG, the methylation difference needs to be ≥ 25%, and q ≤ 0.05; 2). For CHH, the difference needs to be ≥ 15%, and q ≤ 0.05; 4). For all other C, the methylation difference needs to be ≥ 20%, and q ≤ 0.05. The thresholds for methylation differences between cytosine context is based on the degree of methylation (CG and CHG highly methylated), and the degree of being noisy (CHH is more noisy than others are). As CHH methylation starts low, a 25% change (like CG/CHG) would be unrealistically large and would miss many real biological changes; thus, it requires a lower effect-size threshold (e.g., 15%).

Differentially methylated regions (DMRs) for each sequence context (CG, CHG and CHH) between two samples were identified using methylkit (v 1.4.1) [56] with the following stringent criteria. For a candidate DMR, it must have more than five methylated cytosines in at least one sample; and more than ten reads covering each cytosine, and more than four reads for supporting a methylated cytosine. DMR length is between 40 bp and 10 kb; and the distance between adjacent methylated cytosines should be < 200 bp and the fold change of the average methylation level should be > 2. Pearson’s chi-square test (χ2) value should be *P* ≤ 0.05. The detected DMRs overlapping any genes or 2 kb gene-flanking regions (upstream or downstream) were kept for further analyses. Differential DNA methylation between the two samples (pair of light conditions: R10 VS RFR10, R10 VS R80, RFR10 VS RFR80, and R80 VS RFR80) at each locus was determined using Pearson’s chi-square test (χ2) in methylKit (v 1.4.1) [56]. The functional enrichment analyses of genes affected by DMRs/DMCs followed the same procedures as that for differentially expressed genes above.

### Accession numbers

Illumina reads of all samples have been submitted to the Sequence Read Archive at the National Center for Biotechnology Information (NCBI SRA, http://www.ncbi.nlm.nih.gov/sra). Stranded RNA-seq data are available under BioProject accession PRJNA1395678 (direct link: https://www.ncbi.nlm.nih.gov/sra/PRJNA1395678), and methylome data are available under BioProject accession PRJNA1435609 (direct link: https://www.ncbi.nlm.nih.gov/sra/PRJNA1435609). All data are publicly available immediately.

### Data availability

The raw sequence data generated in this study have been deposited in the NCBI Sequence Read Archive (SRA) under the BioProject accessions PRJNA1395678 (RNA-seq) and PRJNA1435609 (methylome). All data are publicly available.

## Results

### Growth and photosynthesis of Norway spruce seedlings in response to different light conditions

**Fig. 1:**
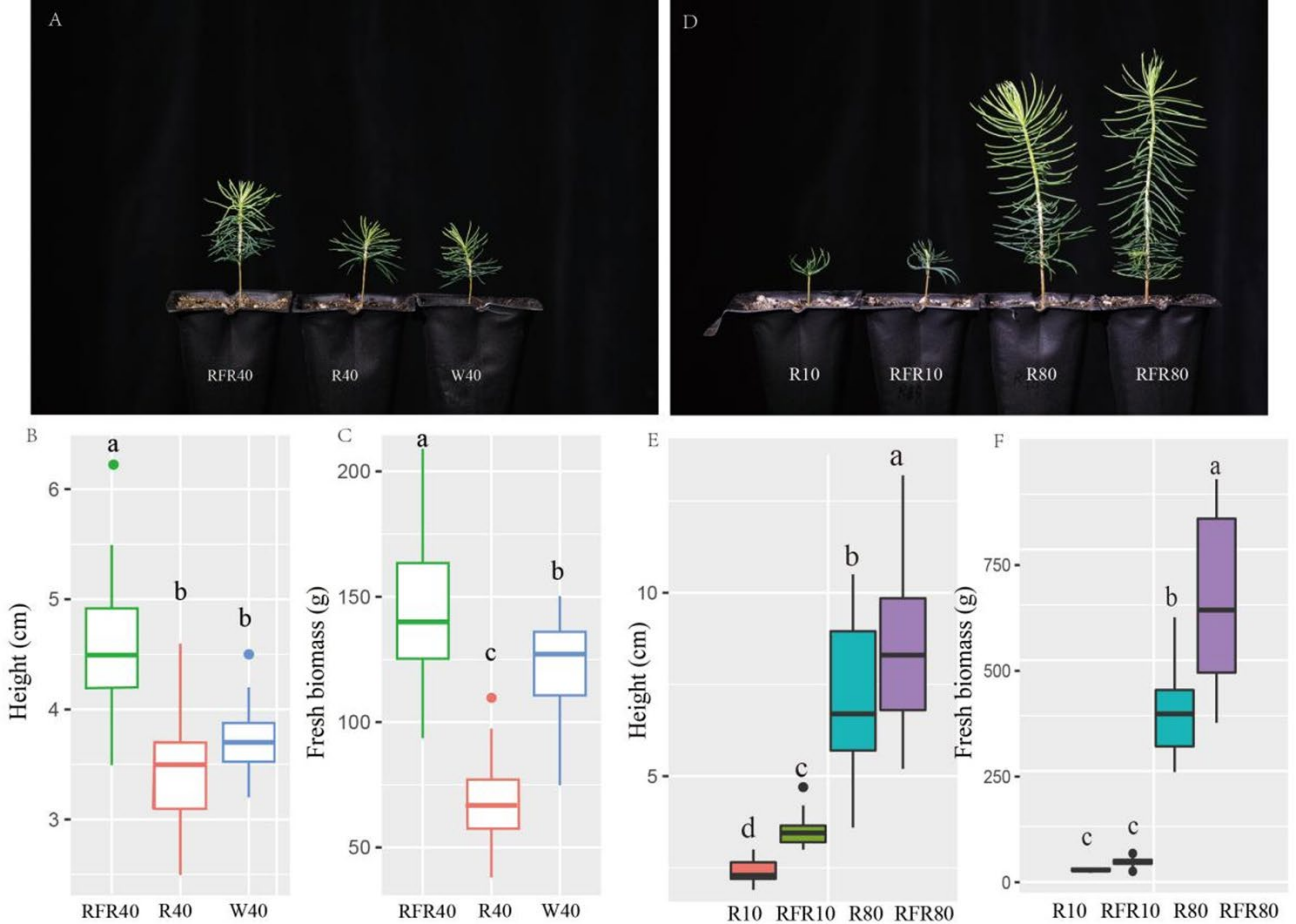
Growth of Norway spruce seedlings in response to different light quality and intensity treatments: Panels **A** and **D** show pictures of representative plants, panels **B** and **E** show plant heights, and panels **C** and **F** show fresh biomass. Where R40, monochrome red light at 40 µmolm^−2^s^− 1^, RFR40, 7/8 monochrome red light plus 1/8 far-red light at 40 µmolm^−2^s^− 1^, W40, white light at 40 µmolm^−2^s^− 1^. R10 and R80, monochrome red light at 10 µmolm^−2^s^− 1^ and at 80 µmolm^−2^s^− 1^, RFR10 and RFR80, 7/8 monochrome red light plus 1/8 far-red light at 10 µmolm^−2^s^− 1^ and at 80 µmolm^−2^s^− 1^. Lower case letters denote significant differences between treatment means using Duncan’s test

The effects of light quality (a mix of red and far-red lights, RFR40, monochrome red light, R40, and white light, W40) at constant light intensity (40 µmolm^−2^s^− 1^) on growth and photosynthesis of seedlings were significant (Table 1). Seedlings grown under RFR40 exhibit larger values for height and fresh biomass than red (R40) or white (W40) light alone (Fig. 1).Similarly, root collar diameter, the longest root length, aboveground biomass and below ground biomass as well as most fluorescence parameters were significantly higher in RFR40 than R40 treatment (data not shown); suggesting that light quality affected both growth and photosynthetic efficiency of Norway spruce seedlings.

**Table 1:** ANOVA results (DF = degrees of freedom, MS = meanquare and P = significance level) of growth responses of Norway spruce seedlings to different light quality treatments at 40 µmolm^−2^s^− 1^

Variance source	Height			Fresh biomass		
	DF	MS	*P* value	DF	MS	*P* value
Light quality	2	6.47	0.001	2	27362.89	0.001
Error	12	0.27		621.39		

To see whether the above observation was dependent on the light intensity, we conducted Two-way ANOVA using light intensities of 10 µmolm^−2^s^− 1^ and 80 µmolm^−2^s^− 1^ and light quality (R and RFR). The results showed that the height of seedlings grown under far-red treatments (RFR10, RFR80) was 3.53 cm and 8.38 cm, respectively, which were 1.46 and 1.19 -fold than that of far-red-light-free treatments (R10, R80) at the same light intensity (Fig. 1D and E, Table 2, *P* < 0.01). Similar trends were observed for fresh biomass (Fig. 1F, Table 2, *P* < 0.01). With this experimental setting, how light intensity influenced growth was also examined. As shown in Fig. 1E and F, the seedling height and the fresh biomass were all significantly larger (*P* < 0.01) in high light intensity (R80, RFR80) than in low intensity (R10, RFR10). There were interaction effects between light intensity and quality on the growth of Norway spruce seedlings (Fig. 1). Fresh biomass differed between R80 and FRR80, but not between R10 and RFR10 (Fig. 1F).

**Table 2:** ANOVA results for the effects of light intensity and quality on plant growth of Norway spruce seedlings

Variance source	Height			Fresh biomass		
	DF	MS	*P* value	DF	MS	*P* value
Light quality	1	37.84	0.001	1	372615.05	0.001
Light intensity	2	217.29	0.001	2	2593943.47	0.001
Light quality × Light intensity	2	0.14	0.001	2	147147.46	0.001
Error	294	1.67		99	9145.67	

### DNA methylation under different light quality and intensity

**Fig. 2:**
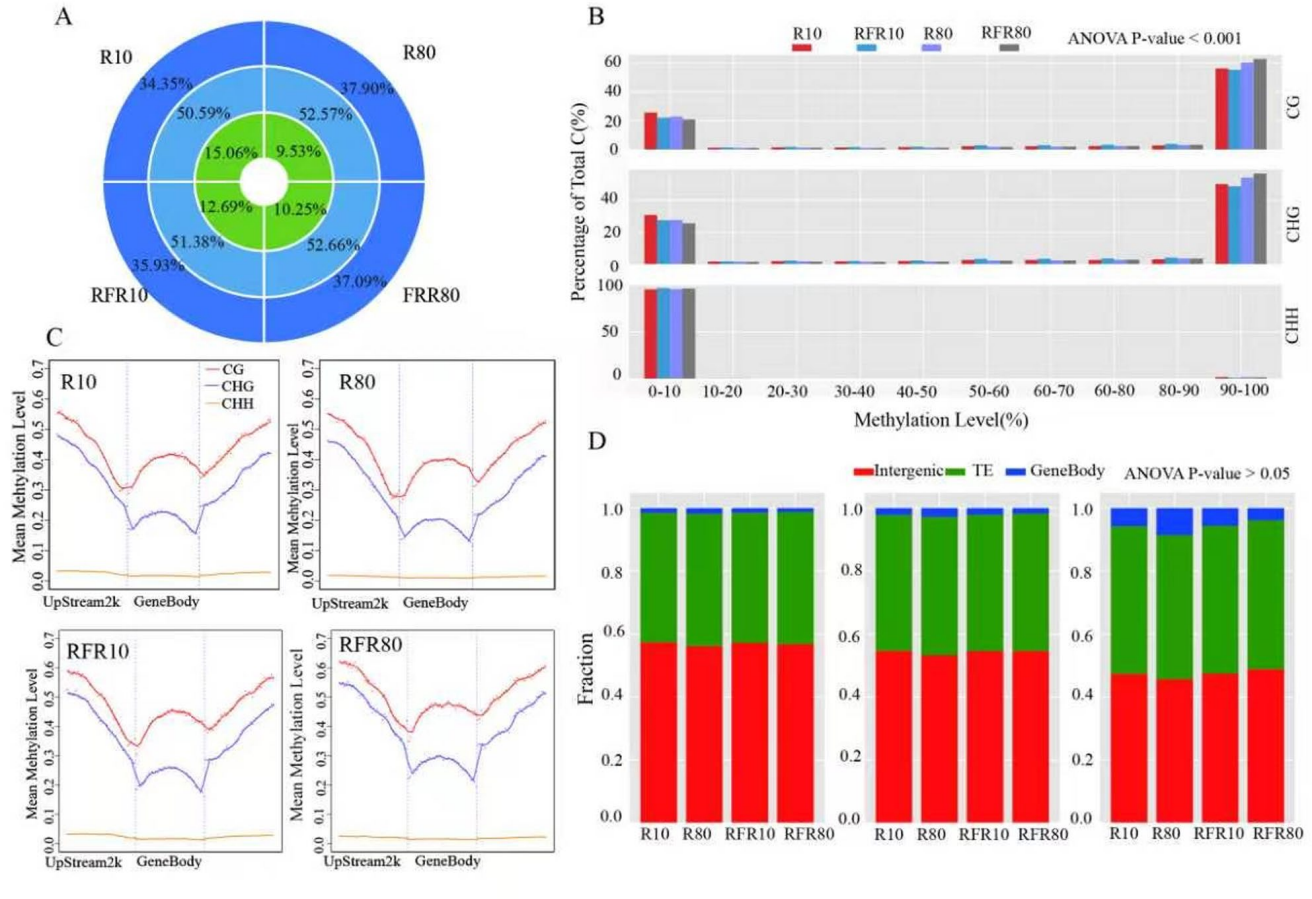
DNA methylomes of *Picea abies* under different light conditions (R10, R80, RFR10, and RFR80). Panel **A**: The percentage of methylated cytosines identified in each condition for each sequence context where blue color denotes the percentage in the CG context, light blue in the CHG context, green in the CHH context. Panel **B**: Distributions of the methylation levels in each sequence context for *P. abies* (ANOVA test, *P* < 0.001) where the x-axis indicates bins of methylation levels, and the y-axis represents the percentage of total cytosines in each bin. Panel **C**: Distributions of methylated cytosine sites among genomic sequence features (gene body, transposon element, and intergenic region) in each cytosine context for *P. abies* for the four light conditions (ANOVA test, *P* > 0.05). Panel **D**: The methylation profiles of cytosines along the length of protein-coding genes (Genes) and transposon elements (TEs). The flanking regions are the same 2 Kb lengths as the genes bodies (ANOVA test, *P* < 0.001). Treatments were as described in Fig. 1

For the four treatments (R10, RFR10, R80, and RFR80) that showed significant effects on growth and photosynthesis, the total numbers of cytosines covered by at least one read were 2.795 billion, 3.477 billion, 3.078 billion, and 3.394 billion, respectively. These represented 14–18% of all cytosines in *Picea abies* genome: 14.3% by R10, 17.7% by RFR10, 15.7% by R80 and 17.3% by RFR80. Among them, about half were in the CHG context, with a smaller proportion in the CG context, and the lowest proportion in CHH context. Notably, with changing treatment conditions, CG proportions increased from 34.35% in R10 to 37.90% in R80, and from 35.93% in RFR10 to 37.09% in RFR80. Conversely, CHH proportions decreased from 15.06% in R10 to 9.53% in R80, and from 12.69% in RFR10 to 10.25% in RFR80 (Fig. 2A). The distributions of cytosine methylation levels varied among cytosine contexts. For mCG and mCHG sites, nearly 60% sites were highly methylated with methylation levels > 90%, and 20% sites were lowly methylated with methylation levels ~ 10%; by contrast, all mCHH sites exhibited lower methylation levels (Fig. 2B; ANOVA test, *P* < 0.001).

There was no difference in the fractions of methylated cytosines in intergenic regions, transposons and gene bodies in all three cytosine contexts (Fig. 2C; ANOVA test, *P* > 0.05). The methylation levels in the CG and CHG contexts were in the order RFR80 > RFR10 > R10 > R80 in both gene body and flanking regions (Fig. 2D), which was based on the methylation profiles along protein-coding genes and their flanking regions (see Materials and Methods for details). While in the CHH context, the order is R10 = RFR10 > RFR80 > R80 (Fig. 2D). The methylation levels in transposable elements (TEs) followed a similar order (among treatments) as in protein coding genes, though no much difference was observed between the TE body and the flanking regions (Fig. 2D).

**Fig. 3:**
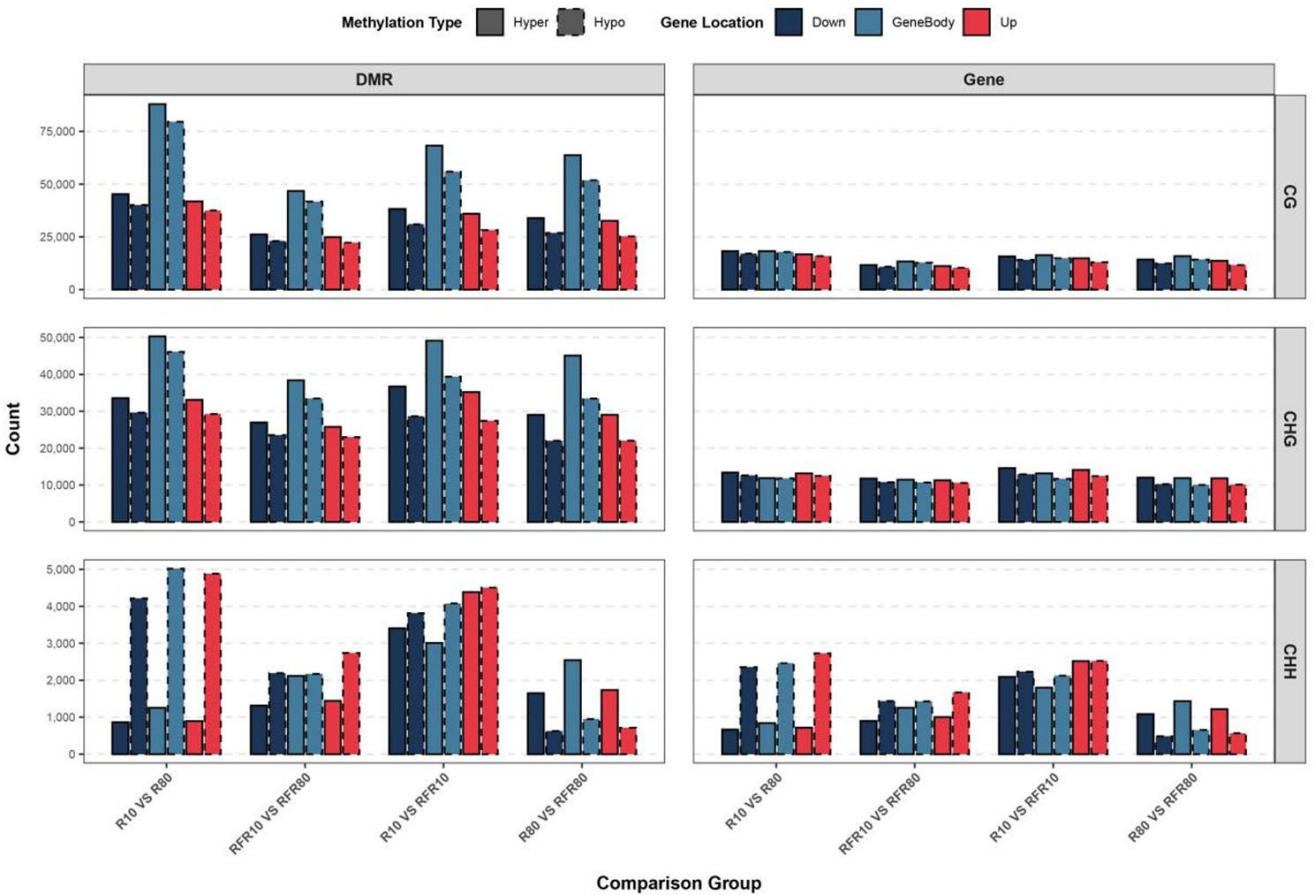
Comparison of the number of differentially expressed regions (DMRs) and the genes located in the DMRs (downstream, gene body and upstream) by methylation type (Hypo- and hyper-methylated) for pairs of light conditions: R10 vs. R80, RFR10 vs. RFR80, R10 vs. RFR10, and R80 vs. RFR80. The gene location up and down represents upstream over 2 k and downstream below 2 k Note that differentially expressed genes were detected using EdgeR with the requirement of |log2FC|>1, FDR < 0.05, Treatments were as described in Fig. 1

To further characterize the methylation differences among treatments (R10, R80, RFR10 and RFR80), we identified differentially methylated regions (DMRs, supplemental Fig. 1). There are more DMRs in the CG and CHG contexts than in the CHH context, but this might be expected because more cytosines were detected in the former two contexts in samples (Fig. 3). In the CG and CHG contexts, the number of hypo- and hyper-methylated regions were similar among all comparisons of light treatments, but in the CHH context, the numbers of hyper- and hypo-methylated DMRs varied among comparisons of light treatments (Fig. 3). For instance, in the comparison of R80 vs. RFR80, there were more hyper-methylated DMRs than hypo-methylated ones in the CHH context, even though the overall methylation level in R80 was lower than that in RFR80 (Fig. 2D).

The functional enrichment of the DMR-associated genes based on the GO annotation was then examined. As shown in Supplemental Table 1, effects of light intensity showed that both R10 vs. R80 and RFR10 vs. RFR80 centered on core regulatory processes including shoot system development, light response, and energy metabolism (carbon fixation). Notably, the RFR10 vs. RFR80 group uniquely activated DMRs associated with reproductive shoot development. For light quality effects, R10 vs. RFR10 and R80 vs. RFR80 shared key regulatory targets such as shoot apical meristem specification and regulation of photomorphogenesis. However, the R80 vs. RFR80 group specifically induced DMRs linked to negative regulation of photomorphogenesis and short-day photoperiodism, reflecting a dose-dependent effect of light intensity on light quality responses—plants require more refined epigenetic regulation to adapt to spectral changes under low light conditions. Regarding functional divergence of DMR contexts, CG-DMRs primarily regulated time-related processes (e.g., photoperiodism, circadian rhythm), CHG-DMRs focused on energy metabolism (e.g., carbon fixation, dark reaction of photosynthesis), and CHH-DMRs specialized in stress and hormone pathways (e.g., abscisic acid response, UV-C response). These three DMR contexts collectively formed an epigenetic regulatory network for plant adaptation to light environment changes, and different combinations of light intensity and quality selectively activated specific DMR contexts, ultimately driving differential plant responses in growth, signal perception, and environmental adaptation.

### Gene expression profiles under light treatments

**Fig. 4:**
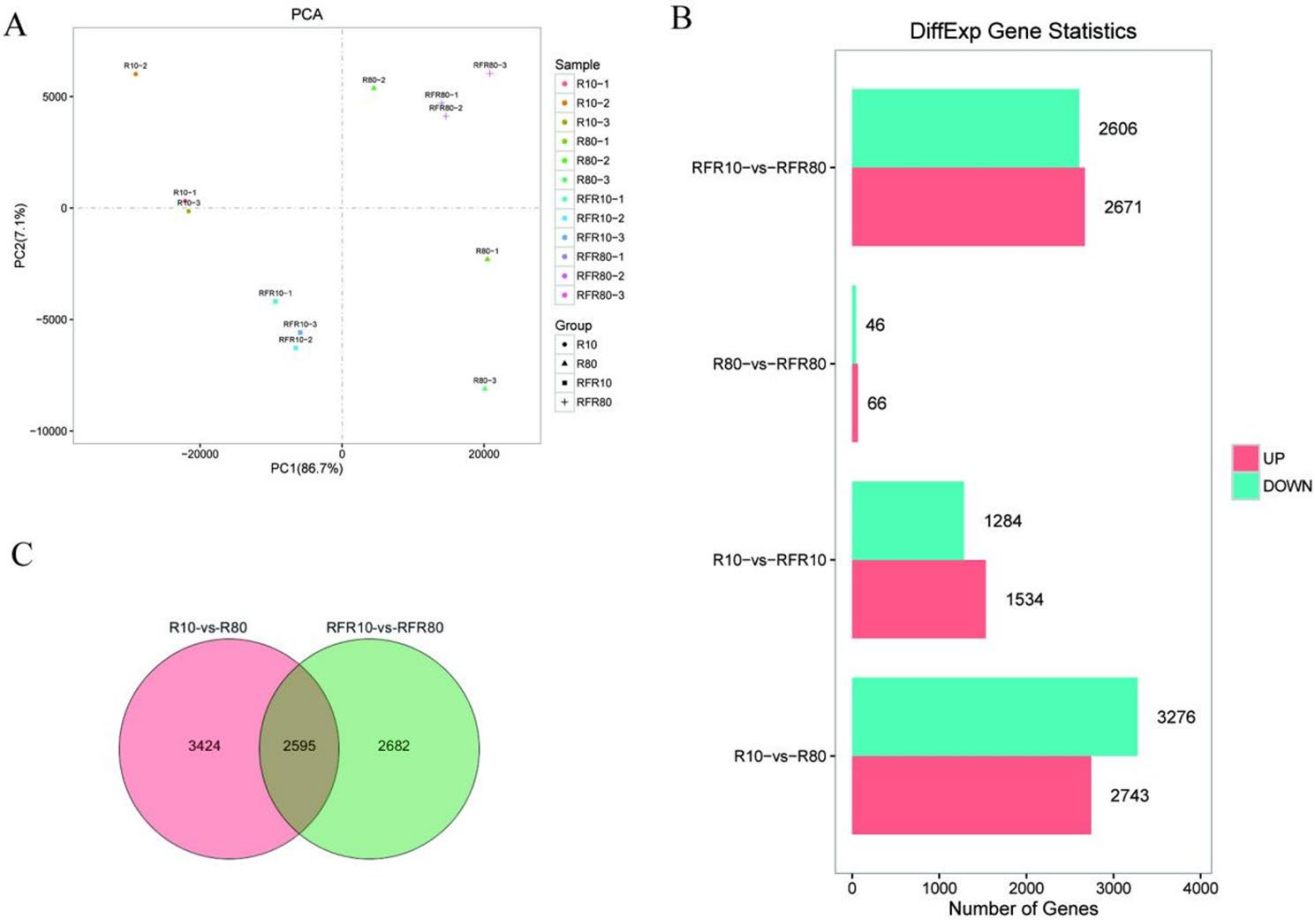
Differentially expressed genes in response to different light treatments. Panel **A**: PCA analysis; Panel **B**: Differences in gene expression (FDR < 0.05 and |log_2_FC|>1) between groups of light treatments (R10 vs. R80, RFR10 vs. RFR80, R10 vs. RFR10, and R80 vs. RFR80). Panel **C**: Venn diagram of differentiated genes between R10 VS R80 and RFR10 VS RFR80. Treatments were as described in Fig. 1

Next, the gene expression under the above four treatments were measured using RNA-seq, with three independent replicates per treatment, yielding a total of 12 libraries. On average, each library generated 58 million paired-end reads and 66.7% of them were uniquely mapped to *P. abies* genome (Supplemental Table 2). With these reads, 43,480 known genes were confirmed and 6227 new genes were discovered (see Supplemental Table 3). As expected, the replicates from the same treatment were clustered together according to the transcriptomes (Fig. 4A). The numbers of differentially expressed genes varied among treatment comparisons (Fig. 4B). The largest number of differentially expressed genes were detected in the comparison of R10 vs. R80 (2743 upregulated and 3276 downregulated), and the smallest were detected in the comparison of R80 vs. RFR80 (66 upregulated and 46 downregulated). Further, about 50% of differentially expressed genes were the same in the comparisons of R10 vs. R80 and RFR10 vs. RFR80 (Fig. 4C).

**Fig. 5:**
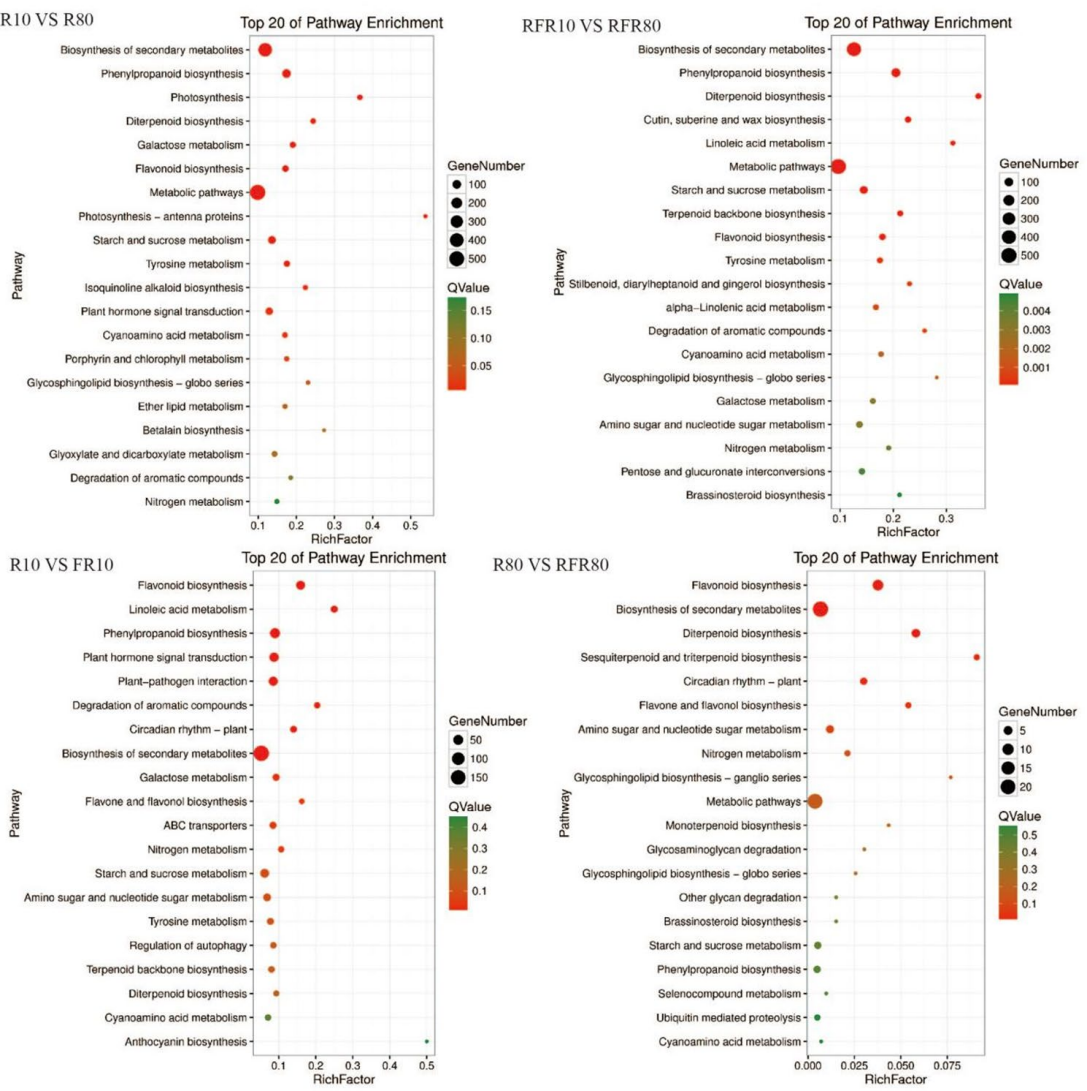
KEGG pathway for comparison of pairs of light treatments (R10 vs. R80, RFR10 vs. RFR80, R10 vs. RFR10, and R80 vs. RFR80). Rich Factor (RF) is the ratio of the number of transcripts in the differentially expressed transcripts in this pathway entry to the total number of transcripts in all the transcripts in this pathway entry, The larger the RF, the higher the degree of enrichment. Treatments were as described in Fig. 1

As shown in Fig. 5, many KEGG pathways frequently stood out in the four treatment comparisons, such as biosynthesis of secondary metabolites, metabolic pathways, starch and sucrose metabolism, and so on, suggesting that light change triggered many metabolic activities. When comparing far-red light treatment to corresponding monochrome red light (R10 vs. RFR10, R80 vs. RFR80), the pathway circadian rhythm popped up. In the comparison of R10 vs. R80, genes related to photosynthesis-antenna proteins popped up too, including *Lhca1*, *Lhcb1*, etc. (Supplemental Table 4). In addition, the differentially expressed genes in other categories, including photosynthesis (Supplemental Table 5), circadian rhythm (Supplemental Table 6), and hormone signal transduction (Supplemental Table 7) were also observed. Some notable differentially expressed genes were the two *PetC* genes of Cytochrome b6/f complex and the b gene of F-type ATPase in the comparisons of R10 VS R80 and RFR10 VS RFR80. These three photosynthesis genes were all upregulated under the higher light intensity treatments regardless of light quality. Another four photosynthesis genes (PSII, *PsaA*, *PsaB*; PSII, *PsbB*; F-type ATPase, Alpha) were also upregulated when comparing the RFR10 to the RFR80 treatment and comparing the RFR10 to R10.

### Correlation between DNA methylation and gene expression changes

**Fig. 6:**
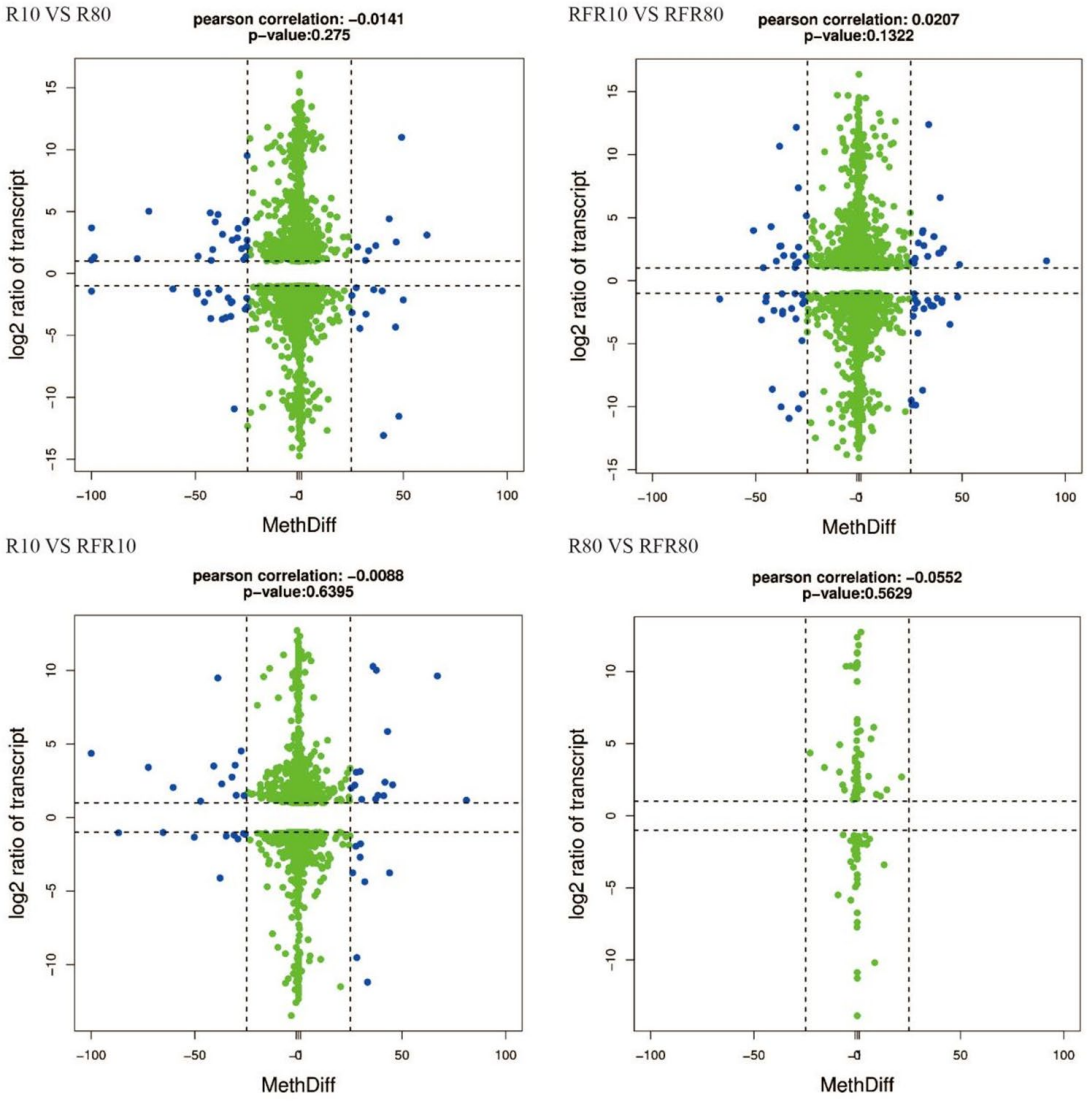
Scatter plot of DNA methylation changes and gene expression changes for comparison of pairs of light treatments: R10 versus R80, RFR10 versus RFR80, R10 versus RFR10, and R80 versus RFR80. Green dots correspond to loci with no significant DNA methylation difference (MethDiff near 0), representing the bulk of the genome where methylation levels remain stable between comparison groups. Their dense distribution around MethDiff = 0 illustrates the overall epigenetic stability of the studied system. Blue dots represent loci with significant DNA methylation differences (absolute MethDiff values exceeding our predefined threshold), which are the candidate epigenetic regulatory sites we focused on. This small subset of dynamically methylated loci and their expression behavior facilitated the identification of potential methylation-mediated regulatory events despite the overall weak linear correlation. Treatments were as described in Fig. 1

There was no correlation between the numbers of regions changed in DNA methylation and in gene expression (Supplemental Table 8). In the comparison of R10 vs. R80, 3.1% of gene body hypo-methylated genes and 2.8% of gene body hyper-methylated genes were upregulated, and these proportions were slightly smaller than that (3.6%) of the genes whose methylation did not change. Similar pattern was observed for gene flanking regions and other comparisons. This was further reinforced by directly comparing the methylation change and gene expression fold changes, where the dense distribution of loci with non-significant methylation around MethDiff = 0 and a small number of loci with significant methylation illustrates the overall epigenetic stability of the studied system (Fig. 6).

**Fig. 7:**
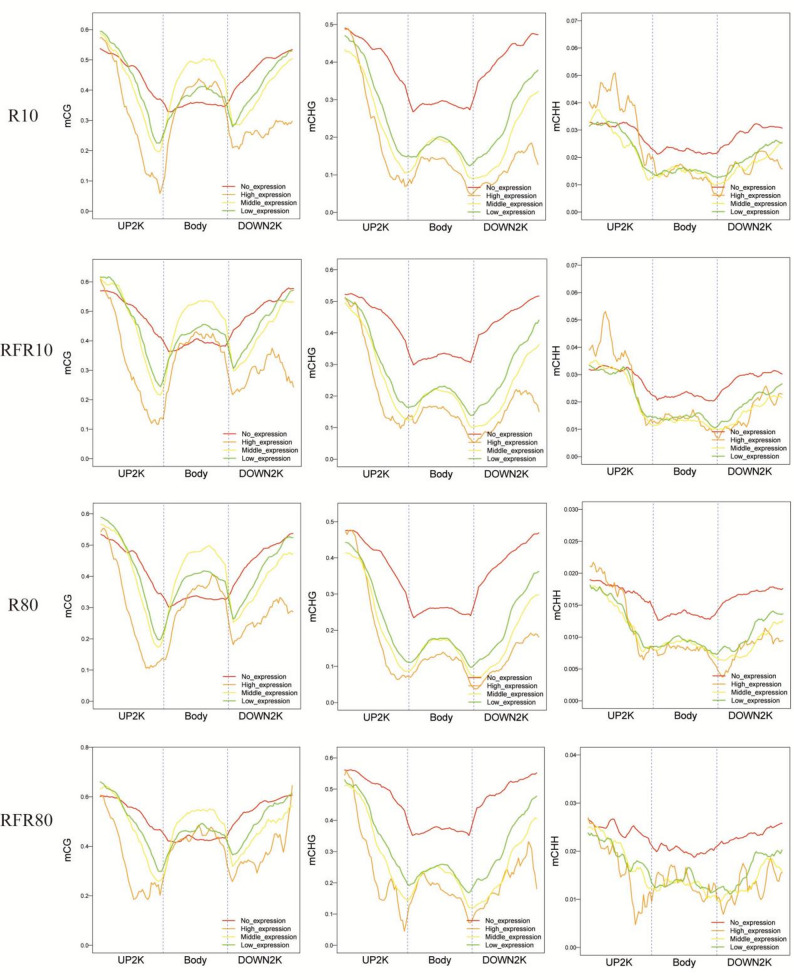
Correlation between DNA methylation and transcription. Expressed genes were divided into four groups based on expression level, including no expression (RPKM ≤ 1), low expression (1 ≤ RPKM ≤ 10), middle expression (10 ≤ RPKM ≤ 100) and high expression (RPKM > 100). Each group has a similar number of genes. Then the average methylation profiles are computed and plotted in each light condition and cytosine context. UP2K, body and Down2K represent the gene locations, which are upstream flank region, gene body and downstream flank region, respectively. Treatments were as described in Fig. 1

However, the correlation between DNA methylation and gene expression showed that the CG methylation level in the gene flanking regions was negatively correlated to gene expression level (Fig. 7). Likewise, the methylation level in both gene body and flanking regions in the CHG and CHH contexts showed a negative relationship with gene expression; though in the CHH context, the methylation difference among low, medium, and high expression groups were negligible. In contrast, the CG methylation level in gene body was moderately positively correlated with gene expression level.

**Fig. 8:**
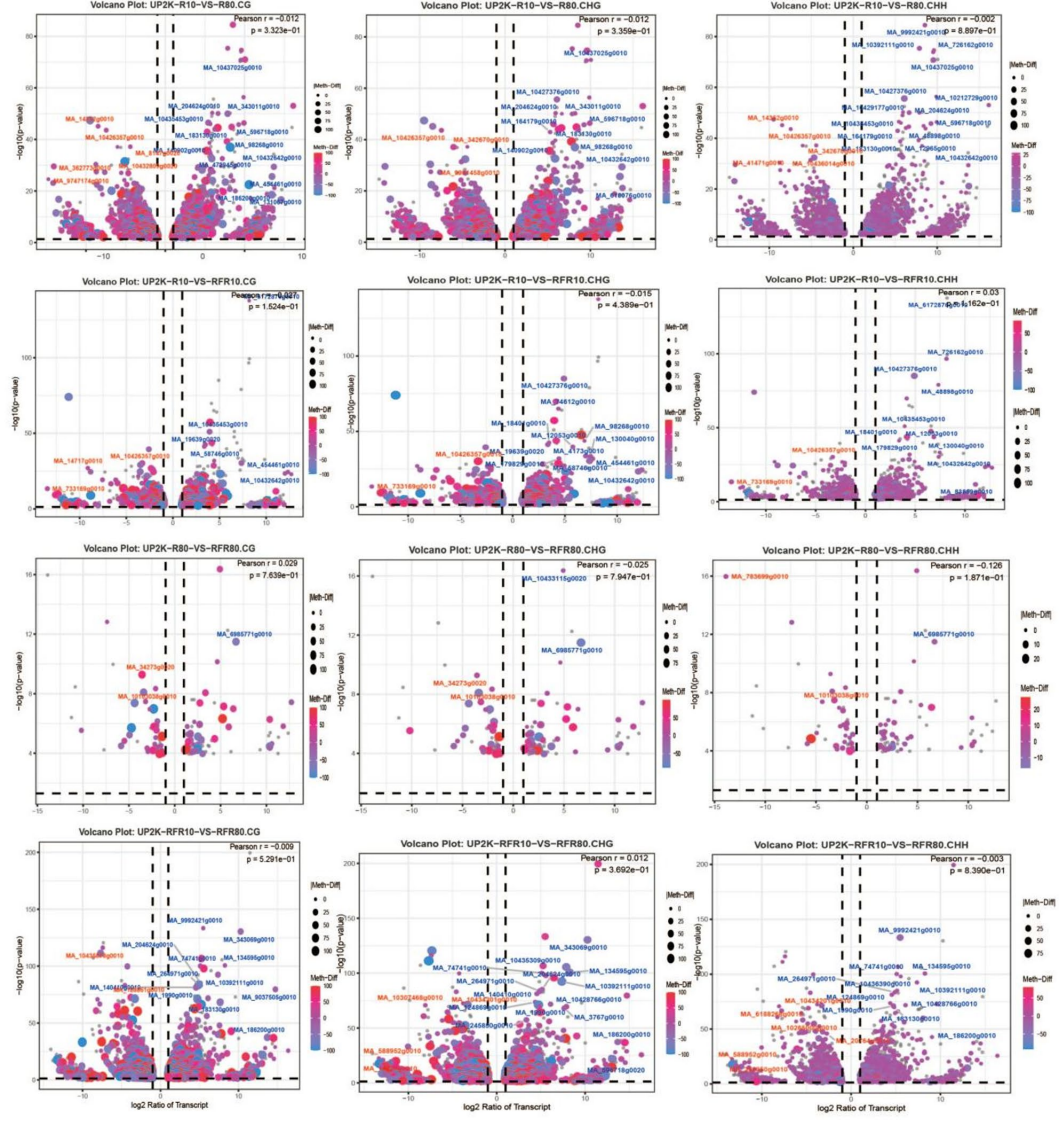
The CG, CHG and CHH modification region of the 2-kb upstream region of genes with a significant negative correlation between methylation and transcription among the four treatment comparison groups for R10 vs. R80, RFR10 vs. RFR80, R10 vs. RFR10, and R80 vs. RFR80. Treatments were as described in Fig. 1

Thus, we focused on the CG, CHG and CHH modification region of the 2-kb upstream region of genes with a significant negative correlation between methylation and transcription among the four comparison groups (Fig. 8). Some genes showed similar methylation and transcriptional change trends in the two comparison groups (R10 vs. R80 and RFR10 vs. RFR80). In photosynthesis - related pathways, when light intensity decreased (e.g., from R80 to R10 or from RFR80 to RFR10), genes such as the chlorophyll a/b - binding protein gene (*cab* gene) and the ribulose − 1,5 - bisphosphate carboxylase/oxygenase small subunit gene (*rbcS* gene) tended to up - regulate their expression through a hypomethylation state. However, in terms of growth and development - related genes, the auxin response factor gene (*ARF* gene) and the cyclin D3 gene (*CYCD3*) showed increased methylation levels and reduced expression under low light. Similarly, in the light quality comparison groups (R10 vs. RFR10), the *ARF* gene and the *CYCD3* gene also showed high methylation and low transcription in the R10 comparison group without far-red light. Secondary metabolism - related genes were also finely regulated by light intensity, light quality and methylation. The cinnamoyl - CoA reductase gene (*CCR* gene), which is involved in lignin synthesis, was hypomethylated with up-regulated transcription under high - light intensity, R80. However, the chalcone synthase gene (*CHS* gene) is hypomethylated with up-regulated transcription in low-light intensity R10, synthesizing more flavonoids. In the R10 vs. RFR10 group, the leucoanthocyanidin reductase 2 gene (*LAR2*), involved in secondary metabolite synthesis, might exhibit a hypomethylation state under RFR10, promoting the increased synthesis of flavonoids. The 2 - methyl − 3 - buten − 2 - ol synthase gene (*MBO synthase*), which participates in terpenoid synthesis, might also have a reduced methylation level under RFR10, resulting in increased terpenoid synthesis. In the R80 vs. RFR80 group, the gene *MA_6985771g0010*, with protein - binding functions, showed a decreased methylation level and increased expression under RFR80 (with added far - red light) across different methylation forms. Meanwhile, some genes involved in secondary metabolite synthesis, such as the E - alpha - bisabolene synthase gene (*MA_34273g0020*), had an increased methylation level and suppressed transcription under RFR80.

**Fig. 9:**
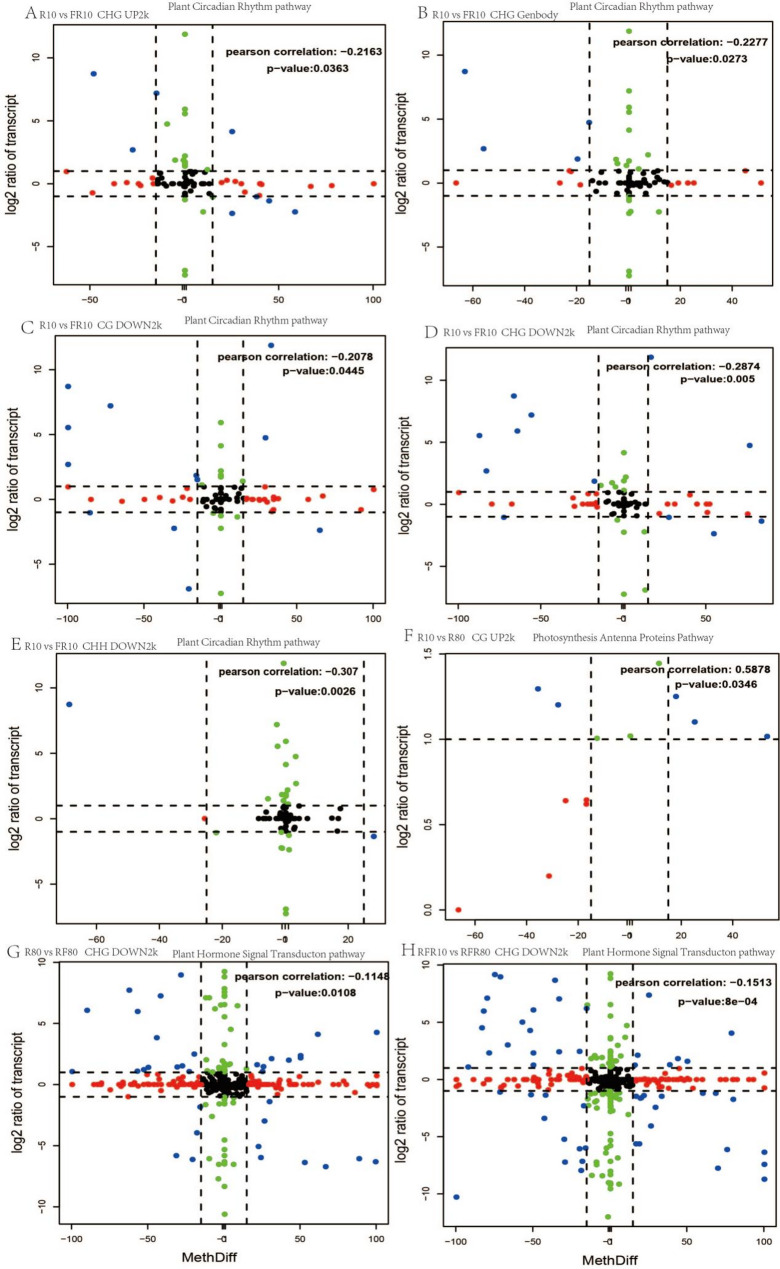
Scatter plot of the genes from the four key pathways (Photosynthesis, Photosynthesis antenna proteins, Plant hormone signal transduction, Plant circadian rhythm) where DNA methylation and transcription levels exhibit a significant correlation—with a distinction made between methylation patterns (CG, CHG, CHH) and methylation positions ( 2-kb upstream region, 2-kb genebody region, 2-kb downstream region) for R10 vs. R80, RFR10 vs. RFR80, R10 vs. RFR10, and R80 vs. RFR80. Treatments were as described in Fig. 1. Blue dots represent genes with positive correlation between methylation difference and transcript log2 ratio (hypermethylation associated with upregulated expression); green dots represent genes with negative correlation (hypermethylation associated with downregulated expression); red dots represent genes with no significant expression change (|log2 ratio| < 1.0); black dots represent genes with no significant methylation difference (|MethDiff| < 10/15, as defined in Methods). Vertical dashed lines indicate the thresholds for significant methylation difference (|MethDiff| ≥ 10/15), while horizontal dashed lines indicate the thresholds for significant transcript expression change (|log2 ratio| ≥ 1.0). **A** Plant Circadian Rhythm pathway, R10 vs. RFR10 group, CHG methylation in the 2-kb upstream region of genes; **B** Plant Circadian Rhythm pathway, R10 vs. RFR10 group, CHG methylation in the gene body; **C** Plant Circadian Rhythm pathway, R10 vs. RFR10 group, CG methylation in the 2-kb downstream region of genes; **D** Plant Circadian Rhythm pathway, R10 vs. RFR10 group, CHG methylation in the 2-kb downstream region of genes; **E** Plant Circadian Rhythm pathway, R10 vs. RFR10 group, CHH methylation in the 2-kb downstream region of genes; **F** Photosynthesis Antenna Proteins pathway, R10 vs. R80 group, CG methylation in the 2-kb upstream region of energy metabolism genes; **G** Plant Hormone Signal Transduction pathway, R80 vs. RFR80 group, CHG methylation in the 2-kb downstream region of genes; **H** Plant Hormone Signal Transduction pathway, RFR10 vs. RFR80 group, CHG methylation in the 2-kb downstream region of genes

To further examine the potential role of the DMRs, we focused on the genes from the four key pathways (Photosynthesis, Photosynthesis antenna proteins, Plant hormone signal transduction, Plant circadian rhythm) where DNA methylation and transcription levels exhibited a significant correlation—with a distinction made between methylation patterns and methylation positions. The results showed significant differences in the Plant Circadian Rhythm pathway for the CHG modification region of the 2-kb upstream region of genes (Pearson correlation = – 0.2163, *P*-value = 0.0363, Fig. 9A) and the gene body (Pearson correlation = – 0.2277, *P*-value = 0.0273, Fig. 9B) for the comparison between the R10 and RFR10 group. Additionally, the three methylation patterns (CG, CHG, CHH) in the 2-kb downstream region of genes also showed significant differences in this group comparison (Fig. 9C, D, E). The genes mainly associated with environmental adaptation including *FT*, *CCA1*, *stilbene synthase* (*STS2*) and *chalcone synthase* and other unidentified genes. In the Photosynthesis Antenna Proteins pathway, comparisons between the R10 and R80 group identified significant differences in CG modifications within the 2-kb upstream region of genes belonging to Energy metabolism (Pearson correlation = 0.5878, *P*-value = 0.0346, Fig. 9F). For the genes mainly associated with the Plant Hormone Signal Transduction pathway, comparisons between the R80 and RFR80 group and the RFR10 and RFR80 group both demonstrated significant differences in CHG modifications within the 2-kb downstream region of genes (Fig. 9G, H). The genes mainly associated with environmental information processing like auxin responsive family genes and other unidentified genes.

We also examined DMRs in the other genes of Photosynthesis, Photosynthesis antenna proteins, and Plant circadian rhythm pathways. Similar to transcriptome results, many genes were uniquely enriched in the comparison of R10 and R80. Specifically, in photosynthesis-antenna proteins, genes encoding light-harvesting complexes of Photosystem I and II (such as *Lhca3*,* Lhca4*,* Lhcb1*) showed increased RNA abundance under R80 treatment compared to R10, accompanied by decreased methylation levels (Supplemental Table 9). In the photosynthesis pathways, genes from the *Psb*, *Psa* and *Pet* families (such as *PsbY*, *PsbP*, *PsaO*, *PsaH*, *PsbW*, *PetH*, and *PsbR*) exhibited decreased methylation in their upstream regions under R80 treatment compared to R10 treatment, alongside increased RNA abundance (Supplemental Table 10). In the plant circadian rhythm pathway, *CK2a* showed hypomethylation under R80 treatment compared to R10, while *PHYA* exhibited increased CG methylation in its upstream region and increased CHG methylation in its gene body under R80 treatment; these methylation changes were coordinated with corresponding expression changes. Coordinated changes between methylation and expression were also observed in other comparisons. In the comparison of RFR10 vs. RFR80, the methylation levels in core photosystem genes (e.g., *PsaA*) and *ATPase* subunit genes decreased in the RFR10 treatment and their expression levels were upregulated (Supplemental Table 10). Similar observations were found in core photosystem genes (e.g., *PsaA*) and circadian rhythm-related genes (e.g., *LHY*, *GI*) when comparing the R10 and RFR10 treatments (Supplemental Tables 10, 11).

## Discussion

### Light intensity and quality affect seedling growth

Our results confirm that both light intensity and quality regulate the development of Norway spruce seedlings. Seedlings grew taller under higher light intensities, and far-red (FR)-containing treatments (RFR10, RFR80); significantly promoted growth compared to monochromatic red light (R10, R80) (Fig. 1). The impact of light on growth might be through increased photosynthesis [57], which we observed in our study. Multiple studies in plant photobiology have investigated the role of light intensity in plant development and revealed that increased light intensity triggers root growth, pigment synthesis and hypocotyl inhibition in both gymnosperms [39, 58, 59] and angiosperms [60, 61]. Theoretically, increased far-red light could increase Pn [61, 62]; through the Emerson enhancement effect: the photosynthetic efficiency of shorter wavelengths (λ < 685 nm) could be improved by the light with longer wavelengths (λ > 700 nm) [62]. Similar results were obtained for Scots pine and Norway spruce where growth under the FR-containing light treatments produced tall seedlings with larger needle dry mass [35].

In addition to the independent effects of light intensity and quality on seedling development, we also found interaction effects (Fig. 1). Similar interaction effects were observed in Scots pine [32, 63] and soybeans [61]. This suggests that the interaction effect may prevail in plant regulation. In fact, the interaction effects may also vary among plant ecotypes. Norway spruce from 62°N can grow normally without far red light treatment [35], but when far red light is deficient, northern populations of Norway spruce need higher light intensities to maintain growth than southern ones [34, 64].

### The methylation patterns differ between CHH and CpG/CHG contexts

Our study revealed that changes in light conditions lead to genome-wide methylation changes, and these changes were determined by both light intensities and qualities and varied among cytosine contexts (CG/CHG vs. CHH). When comparing FR-free conditions (R10 and R80 treatments) with FR-containing ones (RFR10 and RFR80 treatments), the methylation in CG and CHG contexts was lower, but not so in the CHH context (Fig. 2D). Also, when comparing R80 to R10 conditions, the number of hypo-methylated genes in the CHH context were nearly three times higher than that of hyper methylated ones (upstream, gene body and downstream), but the numbers were similar for CG and CHG contexts. These context-specific differences are consistent with a prior study on spruce, where somatic embryogenesis culture cells exhibited lower CG/CHG methylation but higher CHH methylation than needles [65]. These results suggest that the methylation in CHH context responds to environment changes differently from the other two contexts in Norway spruce. As a whole, whole-genome methylation increased in response to far-red light and high light intensity treatment (Fig. 2), indicating that epigenetic modification plays a role in adaptation to light conditions as shown for epigenetic regulation of other environmental stress conditions [66, 67].

The methylation difference in CHH context might be related to its functional difference. It was proposed that CHH methylation does not play a significant role in regulating seed development genes, but behaves as a failsafe mechanism to reinforce transposon silencing [68]. In Norway spruce, the TEs and other repetitive elements account for approximately 70% of the genome [32] compared to ~ 20–25% in *Arabidopsis thaliana* [69] and ~ 60–70% in *Gossypium* species [70], suggesting that CHH methylation is particularly important in gymnosperms than angiosperms. Consistent with this, CHH methylation was higher in TEs than flanking regions (Fig. 2D), and lower in gene bodies than flanking regions (similar to CG/CHG), confirming CHH methylation as a major TE suppression strategy.

While negative correlations between methylation (gene body/promoter) and expression [71] have been reported in upland cotton [72] and *Arabidopsis* [73], rice and citrus showed no genome-wide correlations [74, 75], and some studies have even observed activation of gene expression by methylation [76, 77]. However, another study reveals a weak positive correlation between gene body methylation and expression in *Arabidopsis* [78]. However, in the present study, we found that the methylation-expression relationships vary among genomic regions and the employed approaches. When looking at the averaged methylation profiles, the CG methylation level in gene body was largely positively correlated with gene expression level, while the level in the flanking regions was negatively correlated to gene expression (Fig. 7). However, when examining the number of genes with changed methylation levels and gene expression, no clear correlations were observed (Supplemental Table 8). This discrepancy likely arises because coordinated methylation-expression changes occur in only a subset of genes [78], while most methylation alterations have minimal effects on expression, or some expression changes are independent of methylation [75].

### The methylation and expression changes of some photosynthesis-related genes are coordinated

Methylation primarily affects a small subset of genes, often involved in key metabolic and regulatory pathways [79, 80]. From the perspective of light intensity, when light intensity decreased (e.g., from R80 to R10 or from RFR80 to RFR10), the *cab* gene and the *rbcS* gene may exhibit a hypomethylated state, thereby upregulating their expression to enhance light energy capture and photosynthetic efficiency, helping plants adapt to low-light environments. We observed lower expression and higher methylation for *Lhcb1*, *Lhca3* and *Lhca4* in R10 compared to R80. This is consistent with their roles in light-harvesting complex (LHCs) photosystems [81–83]. It is likely that epigenetic regulation of *LHC* genes through methylation plays a role in plants or dinoflagellate to critical light conditions [25, 84]. In addition, we observed hypermethylation in R10 treatment compared to R80 treatment for low molecular mass proteins such as PsaH, PsaO, PsbP, PsbR, PsbW, PsbY, and PetH, which are located in the inner thylakoid membrane and their roles in photosystem I and II pathways remain to be explored. For growth-related genes, *ARF* and *CYCD3*, exhibited increased methylation and reduced expression under low light, slowing cell division and allowing resource allocation to basic physiological functions—highlighting light intensity’s dominant role in growth regulation.

Those above genes, like *cab* and *rbcS*, and so on, exhibited similar trends of change when comparing R10 vs. R80 and RFR10 vs. RFR80 groups, indicating that light intensity plays a dominant regulatory role. However, some genes exhibited divergent trends, reflecting the interaction between light quality and light intensity. For example, certain genes involved in secondary metabolite biosynthesis, such as the 2-methyl-3-buten-2-ol synthase gene (*MBO synthase*), displayed reduced methylation under low light in the R10 vs. R80 comparison group to promote terpenoid synthesis. In contrast, in the RFR10 vs. RFR80 group, the presence of far-red light altered the methylation pattern, highlighting the influence of light quality on the plant’s response to the light environment. Similar results were observed for European beech where methylation at particular loci was influenced by photoperiod or the local climatic condition [85]. Additionally, auxin response factor genes (*ARF* genes, e.g., *ARF7*), which function in phytohormone signal transduction pathways, exhibited differing trends between the two comparison groups, suggesting that both light quality and intensity jointly regulate plant growth-related signaling pathways.

When exploring how far-red light regulates plant growth, we found that far-red light conditions mainly affected circadian rhythms pathways. By comparing R10 and RFR10 treatments, *LHY*, *GIGANTEA (GI)* and *FT* were the ones hypermethylated in R10 treatment compared with RFR10. *LHY* is an important component of the circadian clock in the model plant *Arabidopsis* and DNA methylation regulates circadian clock [86, 87]. To measure day length, plants require the circadian clock, which regulates internal oscillators [88]. *GI* is a nuclear protein with multiple functions, promoting flowering in long days, sensing light for the circadian clock, and seedling photomorphogenesis under the condition of continuous red light [89]. *FT* affect circadian rhythms and photomorphogenesis, its expression is low in long days and increases substantially in short day [90]. This altered circadian regulation could affect photosynthetic and metabolic pathways as well as overall regulatory networks related to growth and development with environmental adaptation including *FT*, *CCA1*, *stilbene synthase* (*STS2*) and *chalcone synthase.* The study on natural variation of DNA methylation and gene expression in Scots pine populations also revealed that differential DNA methylation and gene expression contribute to local adaptation, thereby enhancing the fitness of Scots pine trees under changing climatic conditions [91].

Far-red light may also affect secondary metabolite synthesis. Taking the leucoanthocyanidin reductase 2 gene (*LAR2*) involved in secondary metabolite synthesis as an example, it may exhibit a hypomethylation state under RFR10 condition, promoting increased flavonoid biosynthesis. Flavonoids possess antioxidant functions that can effectively help plants resist low-light stress, thereby improving growth performance [92]. Additionally, the 2-methyl-3-buten-2-ol synthase gene (*MBO synthase*), which participates in terpenoids synthesis, may show reduced methylation levels under RFR10 conditions, leading to enhanced terpenoids production. This plays a positive role in plant defense and growth regulation, contributing to superior plant growth compared to the state under R10 conditions. Likewise, in the R80 and RFR80 comparison group, for some genes involved in the synthesis of secondary metabolites, such as the *E-alpha-dicyclic sesquimihydrate apenyl synthase* gene, the methylation degree increased and transcription was suppressed under RFR80, suggesting that plants adjusted their secondary metabolic pathways to adapt to new light quality. This suggests that far - red light plays an important role in regulating plant growth and stress responses at the epigenetic level.

## Conclusions

The effects of light intensity and light quality on growth, DNA methylation and gene expression of *P. abies* seedlings were examined. The findings demonstrated that both light intensity and quality affect seedling growth, with the former playing a major role. Changes in light conditions lead to changes in genome-wide methylation, and vary among cytosine contexts (CG/CHG vs. CHH). Whole-genome methylation increased in response to mixture of red and far-red light and high light intensity treatment, indicating that epigenetic modification plays a role in adaptation to light conditions. The changes in light quality and intensity yielded a similar number of DMRs (differentially methylated regions), but light intensity is a major contributor in gene expression changes. There was no significant linear correlation between DNA methylation changes and gene expression changes; however, for some photosynthesis-related genes and circadian rhythms pathway genes, the methylation and gene expression changes are coordinated, and thus these genes can be further studied in the future. As a whole, our study provides a global picture of DNA methylation and gene expression in conifers under different light conditions and paves a foundation for future studies of plant light regulation. As our approach did not employ a multi omics approach, future study to unravel the relationship between genes with changes in methylation and changes in gene expression should focus on a multi omics approach, as methylation is only one regulatory mechanism.

## Supplementary Information

Supplementary Material 1.

Supplementary Material 2.

Supplementary Material 3.

## Data Availability

All raw sequence data are available in the NCBI SRA database under BioProject accessions PRJNA1395678 and PRJNA1435609.
