# Supplementary material for: Response of DNA methylation and gene expression to light treatments in Norway spruce [Picea abies (L.) Karst.]
Source: BMC Genomics. 2026 May 6;27:569. doi: 10.1186/s12864-026-12877-7 (PMC13321527; doi:10.1186/s12864-026-12877-7)
Supplement: Supplementary file 2 — Supplementary Material 2. [file 12864_2026_12877_MOESM2_ESM.docx]

Supplemental Table 1. Biological Process of R10 VS RFR10, R80 VS RFR80, R10 VS R80, RFR10 VS RFR80 for genes covered by DMRs of CG, CHG, and CHH in the GO annotation.

| DMRs | R10 VS R80 | RFR10 VS RFR80 | R10 VS RFR10 | R80 VS RFR80 |
| --- | --- | --- | --- | --- |
| CG | photomorphogenesis (31);  photoperiodism (18);  regulation of shoot system development (57);  regulation of signal transduction (42);  response to light intensity (37);  response to low fluence blue light stimulus by blue low-fluence system (2);  regulation of circadian rhythm (10);  regulation of response to red or far red light (6) | regulation of shoot system development (37);  response to UV (23);  regulation of circadian rhythm (6);  response to low light intensity stimulus (2);  regulation of signal transduction (28) | primary shoot apical meristem specification (9);  response to low fluence blue light stimulus by blue low-fluence system (2);  regulation of circadian rhythm (7);  response to low light intensity stimulus (2) | photomorphogenesis (22);  primary shoot apical meristem specification (8);  response to UV-C (4);  red or far-red light signaling pathway (8);  regulation of circadian rhythm (8);  regulation of shoot system development (40);  response to light stimulus (97);  response to red or far red light (36) |
| CHG | carbon fixation (8);  photomorphogenesis (45);  response to low light intensity stimulus (3);  response to red or far red light (66);  regulation of shoot system development (67) | carbon fixation (6);  photosynthesis, dark reaction (2);  regulation of shoot system development (51);  regulation of signal transduction (42) | carbon fixation (7);  photosynthesis, dark reaction (2);  positive regulation of circadian rhythm (2);  regulation of intracellular signal transduction (17);  regulation of shoot system development (56) | photomorphogenesis (38);  PSII associated light-harvesting complex II catabolic process (6);  response to red or far red light (60);  regulation of shoot system development (62);  response to UV-C (4);  regulation of photomorphogenesis (5) |
| CHH | calcium-mediated signaling (4);  electron transport chain (13);  photoperiodism, flowering (9);  response to starvation (19);  response to abscisic acid (36);  regulation of shoot system development (25);  response to UV-C (2) | regulation of circadian rhythm (4);  reproductive shoot system development (23);  shoot system development (36) | primary shoot apical meristem specification (6);  response to UV-C (2);  response to red or far red light (24);  shoot system morphogenesis (34) | electron transport chain (6);  negative regulation of photomorphogenesis (2);  positive regulation of circadian rhythm (1);  regulation of shoot system development (12);  regulation of response to red or far red light (2);  short-day photoperiodism (1) |

Supplemental Table 2. Summary of the RNA-seq data

| Sample | Total Reads  used to map to the genome | Unmapped Reads (Ratio %) | Unique Mapped Reads (Ratio %) | Multiple Mapped reads (Ratio %) | Mapping Ratio (%) |
| --- | --- | --- | --- | --- | --- |
| R10-1 | 47707684 | 14991835 (31.42) | 32052917 (67.19) | 662932 (1.39) | 68.58 |
| R10-2 | 60878860 | 19631619 (32.25) | 40463229 (66.47) | 784012 (1.29) | 67.75 |
| R10-3 | 62521082 | 20188235 (32.29) | 41520901 (66.41) | 811946 (1.30) | 67.71 |
| RFR10-1 | 57572722 | 18439646 (32.03) | 38457018 (66.80) | 676058 (1.17) | 67.97 |
| RFR10-2 | 55018558 | 17630604 (32.04) | 36724876 (66.75) | 663078 (1.21) | 67.96 |
| RFR10-3 | 59248420 | 19187570 (32.38) | 39319676 (66.36) | 741174 (1.25) | 67.62 |
| R80-1 | 55019368 | 18150925 (32.99) | 36217387 (65.83) | 651056 (1.18) | 67.01 |
| R80-2 | 59642970 | 19224379 (32.23) | 39666579 (66.51) | 752012 (1.26) | 67.77 |
| R80-3 | 61836798 | 19271539 (31.17) | 41800995 (67.60) | 764264 (1.24) | 68.83 |
| RFR80-1 | 58227662 | 18577828 (31.91) | 38956600 (66.90) | 693234 (1.19) | 68.09 |
| RFR80-2 | 58021444 | 18325204 (31.58) | 38992984 (67.20) | 703256 (1.21) | 68.42 |
| RFR80-3 | 59612014 | 19289843 (32.36) | 39618321 (66.46) | 703850 (1.18) | 67.64 |
| Average | 57942298 | 18575769 (32.05) | 38649290 (66.71) | 717239 (1.24) | 67.95 |

Supplemental Table 3. Summary of known and new genes detected from RNA-seq data

| Sample Name | Known Gene No. | New Gene No. | All Gene No. |
| --- | --- | --- | --- |
| R10-1 | 33101 (46.80%) | 5424 | 38525 |
| R10-2 | 34957 (49.42%) | 5458 | 40415 |
| R10-3 | 34230 (48.39%) | 5476 | 39706 |
| RFR10-1 | 34824 (49.23%) | 5572 | 40396 |
| RFR10-2 | 34296 (48.48%) | 5399 | 39695 |
| RFR10-3 | 34467 (48.73%) | 5414 | 39881 |
| R80-1 | 33006 (46.66%) | 5342 | 38348 |
| R80-2 | 34351 (48.56%) | 5405 | 39756 |
| R80-3 | 33675 (47.61%) | 5357 | 39032 |
| RFR80-1 | 34188 (48.33%) | 5540 | 39728 |
| RFR80-2 | 34616 (48.94%) | 5476 | 40092 |
| RFR80-3 | 33952 (48.00%) | 5418 | 39370 |
| In total | 43480 ( 61.47% ) | 6227 | 49707 |

Supplemental Table 4. Differentially expressed genes associated with Norway spruce photosynthesis-antenna proteins under different light conditions “/” indicates no significant accumulation.

| Gene name | Gene id | R10 | RFR10 | R80 | RFR80 | log_2_(R10/R80) | log_2_(RFR10/RFR80) | log_2_(R10/RFR10) | log_2_(R80/RFR80) |
| --- | --- | --- | --- | --- | --- | --- | --- | --- | --- |
| *Lhcb1* | MA_131587g0010 | 10304 | 15744 | 22100 | 19026 | 1.10 | / | / | / |
| *Lhcb2* | MA_10431300g0010 | 1348 | 2221 | 2728 | 2602 | 1.02 | / | / | / |
| *Lhcb4* | MA_129206g0010 | 4302 | 7637 | 10230 | 9824 | 1.25 | / | / | / |
| *Lhcb5* | MA_176279g0010 | 4274 | 6421 | 8652 | 8379 | 1.02 | / | / | / |
| *Lhca1* | MA_10431493g0010 | 1191 | 2382 | 2923 | 3017 | 1.29 | / | / | / |
| *Lhca3* | MA_815900g0010 | 2966 | 4159 | 5953 | 5483 | 1.01 | / | / | / |
| *Lhca4* | MA_65346g0010 | 2567 | 4307 | 5905 | 5089 | 1.20 | / | / | / |

Supplemental Table 5. Differentially expressed genes associated with Norway spruce photosynthesis under different light conditions. “/” indicates no significant difference.

|  | **Gene name** | **Gene ID** | **log_2_(R10/R80)** | **log_2_(RFR10/RFR80)** | **log_2_(R10/RFR10)** | **log_2_(R80/RFR80)** |
| --- | --- | --- | --- | --- | --- | --- |

| photosystem Ⅰ | *PsaA* | MA_10435672g0010 | / | 1.59 | -2.17 | / |
| --- | --- | --- | --- | --- | --- | --- |
|  | *PsaB* | MA_10356500g0030 | / | 1.88 | -2.77 | / |
|  | *PsaD* | MA_9169733g0010 | 1.33 | / | / | / |
|  | *PsaF* | MA_10090427g0010 | 1.53 | / | / | / |
|  | *PsaG* | MA_100910g0020 | 1.11 | / | / | / |
|  | *PsaH* | MA_65259g0010 | 1.32 | / | / | / |
|  | *PsaK* | MA_16054g0010 | 1.16 | / | / | / |
|  | *PsaN* | MA_348255g0010 | 1.11 | / | / | / |
|  | *PsaO* | MA_893g0010 | 1.03 | / | / | / |
| photosystem Ⅱ | *PsbB* | MA_10431364g0010 | / | 1.90 | -3.68 | / |
|  | *PsbO* | MA_8429061g0010 | 2.54 | / | 2.40 | / |
|  | *PsbP* | MA_3005g0010 | 1.04 | / | / | / |
|  | *PsbQ* | MA_218996g0010 | 1.04 | / | / | / |
|  | *PsbR* | MA_282g0010 | 1.19 | / | / | / |
|  | *PsbS* | MA_575444g0010 | 1.47 | / | / | / |
|  | *PsbS* | XLOC_088029 | 1.57 | / | / | / |
|  | *PsbW* | MA_10431532g0010 | 1.24 | / | / | / |
|  | *PsbY* | MA_12201g0010 | 1.29 | / | / | / |
|  | *Psb28* | MA_18349g0010 | 1.79 | / | / | / |
| F-type ATPase | *Alpha* | XLOC_093165 | / | 1.30 | -2.64 | / |
|  | *gamma* | MA_8617095g0010 | 1.04 | / | / | / |
|  | *delta* | MA_657g0010 | 1.34 | / | / | / |
|  | *b* | MA_128169g0010 | 1.73 | 1.14 | / | / |
| Cytochrome b6/f complex | *PetC* | MA_733845g0010 | 1.62 | 1.11 | / | / |
|  | *PetC* | MA_32523g0010 | 1.54 | 1.21 | / | / |
| Photosynthetic electron transport | *PetH* | MA_193431g0010 | 1.43 | / | / | / |

Supplemental Table 6. Differentially expressed genes associated with Norway spruce Circadian rhythm – plant under different light conditions. “/” indicates no significant accumulation.

| Gene name | Gene ID | R10 VS R80 log2(FC) | RFR10 VS RFR80 log2(FC) | R10 VS RFR10 log2(FC) | R80 VS RFR80 log2(FC) |
| --- | --- | --- | --- | --- | --- |
| *PHYA* | MA_6809g0010 | / | -1.02 | / | / |
| *PHYA* | MA_73153g0010 | -2.07 | -1.75 | / | / |
| *PHYB* | MA_195682g0010 | / | -1.58 | / | / |
| *PIF3* | MA_29186g0010 | / | -1.28 | 1.11 | / |
| *TOC1* | MA_71728g0010 | -1.18 | -1.26 | / | / |
| *LHY* | MA_11267g0020 | / | 2.04 | / | / |
| *LHY* | MA_102199g0010 | / | / | 1.53 | / |
| *LHY* | MA_115536g0010 | / | / | 1.38 | / |
| *HY5* | MA_41006g0010 | / | 1.00 | / | / |
| *HY5* | MA_10429552g0010 | / | 2.11 | -2.25 | / |
| *GI* | MA_175520g0010 | / | -2.54 | / | / |
| *GI* | MA_19575g0010 | / | -3.10 | 2.19 | / |
| *FT* | MA_400747g0010 | / | / | 1.88 | / |
| *CK2α* | MA_10436016g0010 | 1.14 | 1.00 | / | / |
| *SPA1* | MA_10426100g0010 | / | -1.19 | / | / |
| *PRR7* | XLOC_062111 | / | / | 1.07 | / |
| *CHS* | MA_401848g0010 | 5.49 | / | 4.14 | / |
| *CHS* | MA_8673616g0010 | / | / | -2.38 | -2.28 |
| *CHS* | MA_827336g0010 | 13.47 | / | 11.86 | / |
| *CHS* | MA_80631g0010 | / | / | -2.24 | / |
| *CHS* | MA_10428225g0010 | -1.23 | -1.66 | / | -1.38 |
| *CHS* | MA_20764g0010 | -1.40 | -2.73 | / | -2.00 |
| *CHS* | MA_5735g0010 | -1.21 | / | -1.37 | / |
| *CHS* | MA_10426264g0020 | / | / | -1.08 | / |
| *CHS* | MA_10426264g0010 | / | / | -1.05 | / |

Supplemental Table 7. Differentially expressed genes associated with Norway spruce plant hormone signal transduction under different light conditions. “/” indicates no significant accumulation.

| **Plant hormone** | **Gene name** | **Gene ID** | **R10 VS R80** | **RFR10 VS RFR80** | **R10 VS RFR10** | **R80 VS RFR80** |
| --- | --- | --- | --- | --- | --- | --- |
| Auxin | *AUXI* | MA_1029359g0010 | -1.4 | / | / | / |
|  | *AUXI* | MA_10432781g0010 | / | 8.7 | / | / |
|  | *AUX/IAA* | MA_10430843g0010 | 2.2 | / | 2.8 | / |
|  | *AUX/IAA* | MA_10430843g0020 | / | / | 1.7 | / |
|  | *AUX/IAA* | MA_62134g0010 | 2.6 | / | 2.4 | / |
|  | *AUX/IAA* | MA_53529g0010 | 2.3 | 2.4 | 1.8 | / |
|  | *GH3* | MA_16777g0010 | -2.9 | -2 | / | / |
|  | *GH3* | MA_10432413g0010 | / | -2.5 | 1.1 | / |
|  | *GH3* | MA_10432413g0020 | / | / | -2.6 | / |
|  | *GH3* | MA_66253g0010 | / | -2.4 | / | / |
|  | *GH3* | MA_212507g0010 | / | 4.1 | / | / |
|  | *ARF* | MA_10432349g0010 | / | -1.4 | / | / |
|  | *SAUR* | MA_13942g0010 | -10.3 | / | / | / |
|  | *SAUR* | MA_43064g0010 | 2 | / | 1.7 | / |
|  | *SAUR* | MA_94838g0010 | 3.1 | / | / | / |
|  | *SAUR* | MA_170671g0030 | -10.1 | -12 | 1.9 | / |
|  | *SAUR* | MA_75109g0010 | / | -10.3 | / | / |
|  | *SAUR* | MA_188586g0010 | -10 | -9.6 | / | / |
|  | *SAUR* | MA_69378g0010 | / | -4.1 | / | / |
|  | *SAUR* | MA_10428249g0010 | / | -2.3 | / | / |
|  | *SAUR* | MA_1020522g0010 | / | -1.8 | / | / |
|  | *SAUR* | MA_117133g0010 | -1.8 | -1.5 | / | / |
|  | *SAUR* | MA_5864g0010 | / | 2.1 | / | / |
| Cytokinine | *CRE1* | MA_10436217g0010 | -2.3 | -1.7 | / | / |
|  | *CRE1* | MA_7398g0010 | -1.8 | -2 | / | / |
|  | *CRE1* | MA_101803g0010 | -1 | / | / | / |
|  | *AHP* | MA_2904g0010 | -2.4 | / | / | / |
|  | *AHP* | MA_10427616g0010 | 1.8 | / | 1.4 | / |
|  | *AHP* | MA_870718g0010 | 3.2 | / | / | / |
|  | *AHP* | MA_130008g0010 | 6.6 | / | / | / |
|  | *B-ARR* | MA_7459g0010 | -1.2 | / | / | / |
|  | *B-ARR* | MA_601142g0010 | / | 3.5 | / | / |
|  | *A-ARR* | MA_104331144g0010 | 1.7 | 1.3 | 1.4 | / |
|  | *A-ARR* | MA_96246g0010 | 3.6 | / | 3.8 | / |
| Gibberellin | *GID1* | MA_9705707g0010 | -2.1 | / | -1.1 | / |
|  | *GID1* | MA_855574g0010 | -1.6 | / | / | / |
|  | *GID1* | MA_131537g0010 | -1.5 | -1.2 | / | / |
|  | *GID1* | MA_269822g0010 | / | 4.7 | / | / |
|  | *GID2* | MA_15269g0010 | -1.6 | -2.3 | / | / |
|  | *GID2* | MA_146085g0010 | -1.6 | -2.3 | / | / |
|  | *GID2* | MA_196364g0010 | -2.4 | -1.8 | -1.5 | / |
|  | *GID2* | MA_10432992g0010 | / | -1.3 | / | / |
|  | *GID2* | MA_92836g0010 | -1.1 | -1.2 | / | / |
|  | *TF* | MA_29186g0010 | / | -1.3 | 1.1 | / |
| Abscisic acid | *PYR/PYL* | MA_9600951g0010 | -2.6 | / | -1.6 | / |
|  | *PYR/PYL* | MA_43111g0010 | -2.5 | / | -1.8 | / |
|  | *PYR/PYL* | MA_10177719g0010 | -2.5 | / | / | / |
|  | *PYR/PYL* | MA_52202g0010 | -2.2 | 3.7 | -5.8 | / |
|  | *PYR/PYL* | MA_74427g0010 | -1.7 | / | -1.7 | / |
|  | *PYR/PYL* | MA_53693g0010 | -1.4 | -1 | / | / |
|  | *PYR/PYL* | MA_287316g0010 | / | / | -2.2 | / |
|  | *PP2C* | MA_26265g0010 | -1.7 | / | -1.2 | / |
|  | *PP2C* | MA_18653g0010 | 1.1 | / | 1.5 | / |
|  | *PP2C* | MA_444738g0020 | -1.7 | / | / | / |
|  | *SnRK2* | XLOC_018141 | -5.6 | -8.2 | / | / |
|  | *SnRK2* | XLOC_004946 | -12.4 | / | / | / |
|  | *SnRK2* | MA_62786g0010 | / | 1.8 | / | / |
|  | *SnRK2* | MA_72136g0010 | -3.2 | / | / | / |
|  | *ABF* | MA_28402g0010 | -1.3 | -1.5 | / | / |
|  | *ABF* | MA_18107g0010 | / | 1.2 | / | / |
| Ethylene | *CTR1* | MA_35694g0010 | -1.3 | / | / | / |
|  | *CTR1* | MA_1005783g0010 | / | 1.9 | / | / |
|  | *MPK6* | MA_104337020g0010 | -1.8 | -1.3 | / | / |
|  | *ERF1/2* | MA_27309g0010 | -3.1 | -5.8 | 1.2 | / |
|  | *EBF1/2* | XLOC_069338 | -10 | / | -10 | / |
|  | *EBF1/2* | XLOC_068288 | / | / | -1.3 | / |
|  | *EBF1/2* | MA_5979847g0010 | -3.4 | / | -3.7 | / |
|  | *EIN3* | MA_53004g0010 | / | / | -1.1 | / |
|  | *EIN3* | MA_10435242g0010 | -1.2 | / | / | / |
|  | *EIN3* | MA_10086718g0010 | -1 | / | / | / |
|  | *EIN3* | MA_20516g0010 | -1 | / | / | / |
| Brassinosteroid | *BRI1* | MA_170g0010 | -1.1 | / | / | / |
|  | *BSK* | XLOC_051981 | -1.7 | / | -1.9 | / |
|  | *TCH4* | MA_10430703g0010 | 2.4 | / | / | / |
|  | *TCH4* | MA_10432086g0010 | 4.3 | 3 | 1.8 | / |
|  | *TCH4* | MA_10429607g0010 | 4.7 | / | 3.4 | / |
|  | *TCH4* | MA_10429607g0020 | 4.8 | 1.2 | 3.2 | / |
| Jasmonic acid | *COI1* | MA_108477g0010 | -1.4 | -1.3 | / | / |
|  | *JAR1* | XLOC_017887 | / | -1 | / | / |
|  | *JAZ* | MA_54375g0010 | -5.9 | / | -11.3 | / |
|  | *JAZ* | MA_132746g0010 | / | 6.1 | -6.8 | / |
|  | *JAZ* | MA_9057085g0010 | / | 4.5 | -5.5 | / |
|  | *JAZ* | MA_10433497g0010 | -2.8 | 3.7 | -5.3 | / |
|  | *JAZ* | MA_9455802g0010 | / | 5 | -5.3 | / |
|  | *JAZ* | MA_155947g0010 | / | 4.3 | -4.7 | / |
|  | *JAZ* | MA_10436587g0010 | -4 | / | -2.2 | / |
|  | *JAZ* | MA_10158949g0010 | / | 2.4 | -2.1 | / |
|  | *JAZ* | MA_6746643g0010 | / | / | -1.2 | / |
|  | *JAZ* | MA_137163g0010 | / | / | -1.2 | / |
|  | *JAZ* | MA_19869g0010 | -3.5 | / | / | / |
|  | *JAZ* | MA_88828g0010 | -2.3 | / | / | / |
|  | *JAZ* | MA_10229741g0010 | / | -8.7 | / | / |
|  | *JAZ* | MA_10430554g0020 | / | 2.5 | / | / |
|  | *COI1* | MA_10436355g0010 | / | -1.6 | / | / |
|  | *COI1* | MA_8264261g0010 | / | -1.6 | / | / |
|  | *MYC2* | MA_10435905g0020 | / | / | -1.4 | / |
| Salicylic acid | *NPR1* | MA_20254g0010 | -2.3 | -2.3 | / | / |
|  | *NPR1* | MA_10436930g0020 | -1.1 | -1.8 | / | / |
|  | *TGA* | MA_130907g0010 | -4.8 | / | -1.6 | / |
|  | *TGA* | MA_101958g0010 | -2 | -2.4 | / | / |
|  | *PR-1* | MA_501572g0010 | / | -5.6 | 3.6 | / |
|  | *PR-1* | MA_1042848g0010 | / | -5.2 | 3.2 | / |

Supplemental Table 8. Correlation between DNA methylation and gene expression changes in Norway spruce seedlings among light conditions, in each cell is the number of genes (up) and fractions (down) in that category.

| R10-VS-R80.CG | Gene body | | | Upstream | | | Downstream | | |
| --- | --- | --- | --- | --- | --- | --- | --- | --- | --- |
|  | RNA abundance up | RNA abundance no change | RNA abundance down | RNA abundance up | RNA abundance no change | RNA abundance down | RNA abundance up | RNA abundance no change | RNA abundance down |
| DNA-meth up | 89  0.028 | 2929  0.93 | 130  0.041 | 43  0.019 | 2068  0.95 | 56  0.026 | 47  0.023 | 1919  0.95 | 58  0.029 |
| DNA-meth no change | 2308  0.036 | 59791  0.925 | 2543  0.039 | 2396  0.036 | 61352  0.92 | 2703  0.041 | 2402  0.036 | 61775  0.92 | 2718  0.04 |
| DNA-meth down | 91  0.031 | 2699  0.916 | 156  0.053 | 49  0.023 | 1999  0.94 | 70  0.033 | 39  0.021 | 1725  0.95 | 53  0.029 |
| Pearson's Chi-squared test | X-squared = 19.862, df = 4, p-value = 0.0005318 | | | X-squared = 41.897, df = 4, p-value = 1.752e-08 | | | X-squared = 33.966, df = 4, p-value = 7.571e-07 | | |

| R10-VS-R80.CHG | Gene body | | | Upstream | | | Downstream | | |
| --- | --- | --- | --- | --- | --- | --- | --- | --- | --- |
|  | RNA abundance up | RNA abundance no change | RNA abundance down | RNA abundance up | RNA abundance no change | RNA abundance down | RNA abundance up | RNA abundance no change | RNA abundance down |
| DNA-meth up | 128  0.027 | 4422  0.93 | 181  0.038 | 68  0.019 | 3373  0.95 | 104  0.029 | 69  0.017 | 3792  0.95 | 113  0.028 |
| DNA-meth no change | 2216  0.036 | 56819  0.92 | 2439  0.040 | 235  0.037 | 58969  0.92 | 2627  0.041 | 2341  0.037 | 58521  0.92 | 2632  0.041 |
| DNA-meth down | 144  0.031 | 4178  0.92 | 209  0.046 | 65  0.020 | 3077  0.95 | 98  0.030 | 78  0.024 | 3106  0.95 | 84  0.026 |
| Pearson's Chi-squared test | X-squared = 17.01, df = 4, p-value = 0.001924 | | | X-squared = 76.818, df = 4, p-value = 8.219e-16 | | | X-squared = 92.956, df = 4, p-value < 2.2e-16 | | |

| R10-VS-R80.CHH | Gene body | | | Upstream | | | Downstream | | |
| --- | --- | --- | --- | --- | --- | --- | --- | --- | --- |
|  | RNA abundance up | RNA abundance no change | RNA abundance down | RNA abundance up | RNA abundance no change | RNA abundance down | RNA abundance up | RNA abundance no change | RNA abundance down |
| DNA-meth up | 10  0.041 | 218  0.90 | 15  0.061 | 8  0.045 | 162  0.92 | 7  0.040 | 4  0.025 | 152  0.95 | 4  0.025 |
| DNA-meth no change | 2421  0.035 | 64051  0.93 | 2740  0.040 | 2436  0.035 | 64134  0.92 | 2771  0.040 | 2448  0.035 | 64213  0.925 | 2774  0.040 |
| DNA-meth down | 57  0.04 | 1150  0.90 | 74  0.060 | 44  0.036 | 1123  0.92 | 51  0.042 | 36  0.031 | 1054  0.92 | 51  0.045 |
| Pearson's Chi-squared test | X-squared = 18.005, df = 4, p-value = 0.001231 | | | X-squared = 0.6792, df = 4, p-value = 0.9539 | | | X-squared = 2.5548, df = 4, p-value = 0.6348 | | |

| R10-VS-RFR10.CG | Gene body | | | Upstream | | | Downstream | | |
| --- | --- | --- | --- | --- | --- | --- | --- | --- | --- |
|  | RNA abundance up | RNA abundance no change | RNA abundance down | RNA abundance up | RNA abundance no change | RNA abundance down | RNA abundance up | RNA abundance no change | RNA abundance down |
| DNA-meth up | 52  0.019 | 2601  0.96 | 30  0.011 | 34  0.017 | 1979  0.97 | 21  0.010 | 28  0.015 | 1852  0.97 | 24  0.013 |
| DNA-meth no change | 1323  0.020 | 63987  0.97 | 953  0.014 | 1351  0.020 | 65060  0.97 | 977  0.014 | 1363  0.020 | 65261  0.97 | 979  0.014 |
| DNA-meth down | 29  0.016 | 1730  0.97 | 31  0.017 | 19  0.014 | 1279  0.97 | 16  0.012 | 13  0.011 | 1205  0.98 | 11  0.009 |
| Pearson's Chi-squared test | X-squared = 4.2973, df = 4, p-value = 0.3673 | | | X-squared = 6.0918, df = 4, p-value = 0.1924 | | | X-squared = 11.559, df = 4, p-value = 0.02095 | | |

| R10-VS- RFR10.CHG | Gene body | | | Upstream | | | Downstream | | |
| --- | --- | --- | --- | --- | --- | --- | --- | --- | --- |
|  | RNA abundance up | RNA abundance no change | RNA abundance down | RNA abundance up | RNA abundance no change | RNA abundance down | RNA abundance up | RNA abundance no change | RNA abundance down |
| DNA-meth up | 59  0.013 | 4416  0.97 | 68  0.015 | 28  0.008 | 3411  0.98 | 28  0.008 | 38  0.010 | 3700  0.98 | 46  0.012 |
| DNA-meth no change | 1299  0.021 | 60886  0.97 | 902  0.014 | 1345  0.021 | 62806  0.96 | 956  0.014 | 1344  0.021 | 62303  0.96 | 947  0.015 |
| DNA-meth down | 46  0.015 | 3016  0.97 | 44  0.014 | 31  0.014 | 2101  0.97 | 30  0.0139 | 22  0.09 | 2315  0.98 | 21  0.009 |
| Pearson's Chi-squared test | X-squared = 16.94, df = 4, p-value = 0.001985 | | | X-squared = 41.061, df = 4, p-value = 2.611e-08 | | | X-squared = 42.34, df = 4, p-value = 1.419e-08 | | |

| R10-VS- RFR10.CHH | Gene body | | | Upstream | | | Downstream | | |
| --- | --- | --- | --- | --- | --- | --- | --- | --- | --- |
|  | RNA abundance up | RNA abundance no change | RNA abundance down | RNA abundance up | RNA abundance no change | RNA abundance down | RNA abundance up | RNA abundance no change | RNA abundance down |
| DNA-meth up | 20  0.028 | 687  0.96 | 11  0.015 | 24  0.025 | 931  0.95 | 23  0.024 | 18  0.023 | 757  0.96 | 11  0.014 |
| DNA-meth no change | 1367  0.020 | 66809  0.97 | 984  0.014 | 1358  0.020 | 66481  0.97 | 971  0.014 | 1363  0.020 | 66785  0.97 | 991  0.014 |
| DNA-meth down | 17  0.020 | 822  0.96 | 19  0.022 | 22  0.023 | 906  0.96 | 20  0.021 | 23  0.028 | 776  0.96 | 12  0.015 |
| Pearson's Chi-squared test | X-squared = 6.2137, df = 4, p-value = 0.1837 | | | X-squared = 10.987, df = 4, p-value = 0.02671 | | | X-squared = 3.4855, df = 4, p-value = 0.4801 | | |

| RFR10-VS-RFR80.CG | Gene body | | | Upstream | | | Downstream | | |
| --- | --- | --- | --- | --- | --- | --- | --- | --- | --- |
|  | RNA abundance up | RNA abundance no change | RNA abundance down | RNA abundance up | RNA abundance no change | RNA abundance down | RNA abundance up | RNA abundance no change | RNA abundance down |
| DNA-meth up | 53  0.026 | 1902  0.94 | 58  0.029 | 31  0.021 | 1418  0.97 | 20  0.014 | 32  0.023 | 1317  0.96 | 23  0.027 |
| DNA-meth no change | 2235  0.033 | 62601  0.93 | 2256  0.033 | 2275  0.033 | 63456  0.93 | 2303  0.034 | 2281  0.033 | 63685  0.93 | 2305  0.034 |
| DNA-meth down | 46  0.028 | 1549  0.95 | 36  0.022 | 28  0.023 | 1178  0.96 | 27  0.022 | 21  0.019 | 1050  0.96 | 22  0.020 |
| Pearson's Chi-squared test | X-squared = 12.406, df = 4, p-value = 0.01457 | | | X-squared = 35.486, df = 4, p-value = 3.69e-07 | | | X-squared = 29.882, df = 4, p-value = 5.173e-06 | | |

| RFR10-VS-RFR80.CHG | Gene body | | | Upstream | | | Downstream | | |
| --- | --- | --- | --- | --- | --- | --- | --- | --- | --- |
|  | RNA abundance up | RNA abundance no change | RNA abundance down | RNA abundance up | RNA abundance no change | RNA abundance down | RNA abundance up | RNA abundance no change | RNA abundance down |
| DNA-meth up | 94  0.026 | 3415  0.95 | 88  0.024 | 42  0.016 | 2517  0.97 | 46  0.018 | 53  0.019 | 2660  0.96 | 59  0.021 |
| DNA-meth no change | 2149  0.033 | 59946  0.93 | 2177  0.034 | 2250  0.034 | 61496  0.93 | 2258  0.03 | 2240  0.034 | 61214  0.93 | 2255  0.034 |
| DNA-meth down | 91  0.032 | 2691  0.94 | 85  0.030 | 42  0.020 | 2039  0.96 | 46  0.022 | 41  0.018 | 2178  0.97 | 36  0.016 |
| Pearson's Chi-squared test | X-squared = 16.964, df = 4, p-value = 0.001964 | | | X-squared = 70.371, df = 4, p-value = 1.895e-14 | | | X-squared = 72.838, df = 4, p-value = 5.71e-15 | | |

| RFR10-VS-RFR80.CHH | Gene body | | | Upstream | | | Downstream | | |
| --- | --- | --- | --- | --- | --- | --- | --- | --- | --- |
|  | RNA abundance up | RNA abundance no change | RNA abundance down | RNA abundance up | RNA abundance no change | RNA abundance down | RNA abundance up | RNA abundance no change | RNA abundance down |
| DNA-meth up | 11  0.026 | 410  0.95 | 9  0.021 | 9  0.030 | 287  0.95 | 5  0.017 | 8  0.028 | 277  0.96 | 4  0.014 |
| DNA-meth no change | 2301  0.032 | 65132  0.93 | 2322  0.033 | 2304  0.033 | 65153  0.93 | 2326  0.033 | 2304  0.033 | 65241  0.93 | 2326  0.033 |
| DNA-meth down | 22  0.040 | 510  0.93 | 19  0.034 | 21  0.032 | 612  0.939 | 19  0.029 | 22  0.038 | 534  0.93 | 20  0.035 |
| Pearson's Chi-squared test | X-squared = 3.7286, df = 4, p-value = 0.444 | | | X-squared = 3.0956, df = 4, p-value = 0.5419 | | | X-squared = 4.253, df = 4, p-value = 0.3729 | | |

| R80-VS-RFR80.CG | Gene body | | | Upstream | | | Downstream | | |
| --- | --- | --- | --- | --- | --- | --- | --- | --- | --- |
|  | RNA abundance up | RNA abundance no change | RNA abundance down | RNA abundance up | RNA abundance no change | RNA abundance down | RNA abundance up | RNA abundance no change | RNA abundance down |
| DNA-meth up | 4  0.001 | 3090  0.998 | 1  0.000 | 0  0 | 2273  1 | 0  0 | 1  0.001 | 1918  0.999 | 2  0.001 |
| DNA-meth no change | 55  0.001 | 65700  0.998 | 41  0.001 | 59  0.001 | 67051  0.998 | 43  0.001 | 57  0.001 | 67573  0.999 | 42  0.001 |
| DNA-meth down | 0  0 | 1843  0.999 | 2  0.001 | 0  0 | 1309  0.999 | 1  0.001 | 1  0.001 | 1142  0.999 | 0  0 |
| Pearson's Chi-squared test | X-squared = 3.3967, df = 4, p-value = 0.4938 | | | X-squared = 4.6458, df = 4, p-value = 0.3256 | | | X-squared = 1.4882, df = 4, p-value = 0.8287 | | |

| R80-VS-RFR80.CHG | Gene body | | | Upstream | | | Downstream | | |
| --- | --- | --- | --- | --- | --- | --- | --- | --- | --- |
|  | RNA abundance up | RNA abundance no change | RNA abundance down | RNA abundance up | RNA abundance no change | RNA abundance down | RNA abundance up | RNA abundance no change | RNA abundance down |
| DNA-meth up | 2  0.001 | 5134  0.999 | 0  0 | 3  0.001 | 3573  0.999 | 1  0 | 0  0 | 3701  1 | 0  0 |
| DNA-meth no change | 56  0.001 | 62597  0.998 | 42  0.001 | 54  0.001 | 64857  0.999 | 42  0.001 | 56  0.001 | 64649  0.998 | 43  0.001 |
| DNA-meth down | 1  0 | 2902  0.999 | 2  0.001 | 2  0 | 2203  0.998 | 1  0 | 3  0.001 | 2283  0.998 | 1  0  0.000 |
| Pearson's Chi-squared test | X-squared = 5.7696, df = 4, p-value = 0.217 | | | X-squared = 0.85358, df = 4, p-value = 0.9311 | | | X-squared = 6.4067, df = 4, p-value = 0.1708 | | |

| R80-VS-RFR80.CHH | Gene body | | | Upstream | | | Downstream | | |
| --- | --- | --- | --- | --- | --- | --- | --- | --- | --- |
|  | RNA abundance up | RNA abundance no change | RNA abundance down | RNA abundance up | RNA abundance no change | RNA abundance down | RNA abundance up | RNA abundance no change | RNA abundance down |
| DNA-meth up | 0  0 | 578  1 | 0  0 | 0  0 | 416  1 | 0  0 | 0  0 | 374  1 | 0  0 |
| DNA-meth no change | 59  0.001 | 69878  0.998 | 44  0.001 | 59  0.001 | 70100  0.998 | 44  0.001 | 59  0.001 | 70146  0.998 | 44  0.001 |
| DNA-meth down | 0  0 | 177  1 | 0  0 | 0  0 | 117  1 | 0  0 | 0  0 | 113  1 | 0  0 |
| Pearson's Chi-squared test | X-squared = 1.1129, df = 4, p-value = 0.8922 | | | X-squared = 0.78314, df = 4, p-value = 0.9407 | | | X-squared = 0.71509, df = 4, p-value = 0.9495 | | |
