# Supplementary material for: Response of DNA methylation and gene expression to light treatments in Norway spruce [Picea abies (L.) Karst.]
Source: BMC Genomics. 2026 May 6;27:569. doi: 10.1186/s12864-026-12877-7 (PMC13321527; doi:10.1186/s12864-026-12877-7)
Supplement: Supplementary file 3 — Supplementary Material 3. [file 12864_2026_12877_MOESM3_ESM.docx]

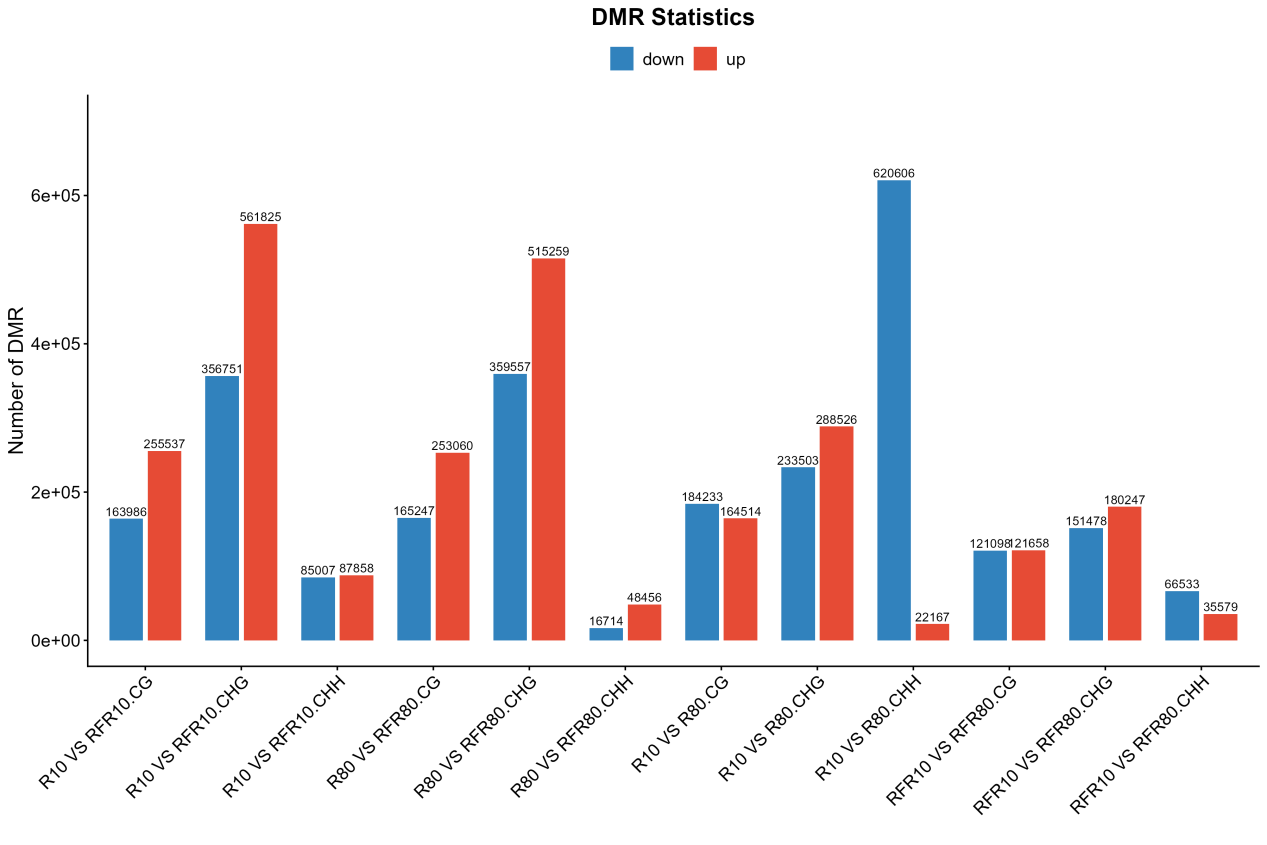


Supplementary Figure 1. Statistics of differentially methylated regions (DMRs) in different cytosine contexts. The number of upregulated (red) and downregulated (blue) DMRs is shown for CG, CHG, and CHH contexts across all comparison groups.
